# Visualizing Vpr-Induced G2 Arrest and Apoptosis

**DOI:** 10.1371/journal.pone.0086840

**Published:** 2014-01-22

**Authors:** Tomoyuki Murakami, Yoko Aida

**Affiliations:** 1 Viral Infectious Diseases Unit, RIKEN, Wako, Saitama, Japan; 2 Laboratory of Viral Infectious Diseases, Department of Medical Genome Sciences, Graduate School of Frontier Science, The University of Tokyo, Wako, Saitama, Japan; Institute of Human Virology, Baltimore, United States of America

## Abstract

Vpr is an accessory protein of human immunodeficiency virus type 1 (HIV-1) with multiple functions. The induction of G2 arrest by Vpr plays a particularly important role in efficient viral replication because the transcriptional activity of the HIV-1 long terminal repeat is most active in G2 phase. The regulation of apoptosis by Vpr is also important for immune suppression and pathogenesis during HIV infection. However, it is not known whether Vpr-induced apoptosis depends on the ability of Vpr to induce G2 arrest, and the dynamics of Vpr-induced G2 arrest and apoptosis have not been visualized. We performed time-lapse imaging to examine the temporal relationship between Vpr-induced G2 arrest and apoptosis using HeLa cells containing the fluorescent ubiquitination-based cell cycle indicator2 (Fucci2). The dynamics of G2 arrest and subsequent long-term mitotic cell rounding in cells transfected with the Vpr-expression vector were visualized. These cells underwent nuclear mis-segregation after prolonged mitotic processes and then entered G1 phase. Some cells subsequently displayed evidence of apoptosis after prolonged mitotic processes and nuclear mis-segregation. Interestingly, Vpr-induced apoptosis was seldom observed in S or G2 phase. Likewise, visualization of synchronized HeLa/Fucci2 cells infected with an adenoviral vector expressing Vpr clearly showed that Vpr arrests the cell cycle at G2 phase, but does not induce apoptosis at S or G2 phase. Furthermore, time-lapse imaging of HeLa/Fucci2 cells expressing SCAT3.1, a caspase-3-sensitive fusion protein, clearly demonstrated that Vpr induces caspase-3-dependent apoptosis. Finally, to examine whether the effects of Vpr on G2 arrest and apoptosis were reversible, we performed live-cell imaging of a destabilizing domain fusion Vpr, which enabled rapid stabilization and destabilization by Shield1. The effects of Vpr on G2 arrest and subsequent apoptosis were reversible. This study is the first to characterize the dynamics of the morphological changes that occur during Vpr-induced G2 arrest and apoptosis.

## Introduction

The human immunodeficiency virus type 1 (HIV-1) accessory protein Vpr has multiple biological functions. In non-dividing cells, such as macrophages, Vpr is important for the nuclear import of the viral preintegration complex and efficient virus replication via proteasome degradation of the endoribonuclease Dicer [1]–[6]. Vpr also regulates splicing [7]–[9], transactivates the viral long terminal repeat (LTR) [10], induces nuclear herniations and cell cycle arrest at G2 phase [11]–[13], and regulates apoptosis, both positively and negatively [14]. The induction of G2 arrest likely plays an important role in efficient viral replication because the transcriptional activity of the HIV-1 LTR is most active in G2 phase [15], [16]. Indeed, the ability of Vpr to cause cell cycle blockade is well conserved among the primate lentiviruses [17], [18]. On the other hand, the regulation of apoptosis by Vpr through direct interaction with the mitochondrion and its ability to alter the balance between pro-apoptotic and anti-apoptotic factors contributes to immune suppression and affects pathogenesis during HIV infection *in vitro* and *in vivo* [14], [19]–[23].

Vpr induces G2 arrest by activating the ataxia telangiectasia-mutated and Rad3-related protein (ATR)-mediated G2/M checkpoint [24], [25]. Caffeine, which inhibits DNA repair by ATR, can reduce Vpr-induced G2 arrest and apoptosis [26]. Moreover, treatment with thymidine, which arrests cells at the G1/S boundary, suppresses Vpr-induced apoptosis [27]. These results indicate that Vpr-induced apoptosis correlates with the induction of G2 arrest. However, some studies have proposed that Vpr-induced apoptosis is independent of G2 arrest. For example, some Vpr point mutants fail to induce apoptosis, although they still induce G2 arrest [28], [29]. In addition, Jacotot *et al*. [19] and Vieira *et al*. [23] reported that recombinant Vpr can bind to the adenine nucleotide transporter protein in the inner mitochondrial membrane and induce apoptosis directly by permeabilizing the mitochondrial outer membrane. This finding indicates that Vpr-induced apoptosis does not require checkpoint activation. Moreover, we reported that a carboxy-terminally truncated form of Vpr, C81, which failed to induce G2 arrest, induced apoptosis via G1 arrest [30], [31]. Thus, Vpr regulates cell cycle progression and apoptosis both positively and negatively; however, the molecular mechanism(s) underlying the regulation of these two processes by Vpr remains obscure. In addition, the morphological dynamics of living cells undergoing Vpr-induced cell cycle G2 arrest and apoptosis have not been studied.

To visualize the dynamics of cell cycle progression, the fluorescent ubiquitination-based cell cycle indicator (Fucci) has been used in HeLa (HeLa/Fucci) cells. HeLa/Fucci cells allow for dual-color imaging; G1-phase nuclei are labeled with monomeric Kusabira Orange and S/G2/M phase nuclei are labeled with monomeric Azami Green. This permits live cells in the G1 to be distinguished from live cells in S/G2/M phases [32]. Recently, Sakaue-Sawano *et al*. developed a new Fucci derivative named “Fucci2” with different fluorescent proteins to provide better color contrast than the original Fucci [33]. HeLa/Fucci2 cells express two fusion proteins: a red monomeric fluorescent protein (mCherry) fused to the truncated form of human Cdt1 (amino acids 30–120) and a monomeric mutant of yellow fluorescent protein (mVenus) fused to Geminin (amino acids 1–110). Both Cdt1 and Geminin express ubiquitination domains. The level of Cdt1 is highest in G1 phase, whereas Geminin accumulates mainly during S/G2/M phase. Therefore, the nuclei of HeLa/Fucci2 cells emit red fluorescence of mCherry-hCdt1 (30/120) in G1 phase and yellow fluorescence of mVenus-hGeminin (1/110) in S/G2/M phases. Thus, HeLa/Fucci2 cells enable the visualization of Vpr-induced G2 cell cycle arrest.

Fluorescence resonance energy transfer (FRET) technology has been widely used to investigate protein–protein interactions. Moreover, it can be effectively used for functional live imaging of caspase activation during apoptosis *in vitro* and *in vivo* [34], [35]. Nagai *et al*. have constructed a high efficiency FRET indicator for caspase-3 activity, SCAT3.1 [36]. It consists of enhanced cyan fluorescence protein (ECFP) as the FRET donor and mVenus as the FRET acceptor, linked by peptides containing the caspase-3 cleavage sequence DEVD. When caspase-3 is activated during apoptosis, SCAT3.1 is cleaved and there is a change in the FRET signal. Therefore, the FRET technique using this SCAT3.1 is applicable to live imaging of caspase-3 activation, which should help understand the dynamics and significance of Vpr-induced apoptosis at the single cell level.

In this study, we first observed that G2 cell cycle arrest occurred in Vpr-expressing HeLa/Fucci2 cells by observing the cells under a CELAVIEW microscope, which allows fully automated image acquisition and data analysis, and permits estimations of DNA content in a large number of cells. Using time-lapse imaging of HeLa/Fucci2 cells, we succeeded in monitoring the dynamics of Vpr-induced cell cycle arrest, which revealed that, although Vpr arrested the cell cycle at G2 phase, it did not induce cell death in cells in S or G2 phase. Similarly, cell death in S or G2 phase was rare in HeLa/Fucci2 cells infected with an adenoviral vector expressing Vpr. Moreover, functional live imaging of caspase-3 activation using cells expressing SCAT3.1 revealed that Vpr-induced apoptosis was caspase-3 dependent. Finally, we demonstrated the reversibility of Vpr-induced apoptosis by using live-cell imaging of cells expressing a destabilizing domain (DD) Vpr fusion protein, which can be rapidly stabilized or destabilized by Shield1, a protein that binds to the DD and protects the entire fusion protein from proteasomal degradation. Importantly, this is the first study to demonstrate a temporal relationship between Vpr-induced cell cycle arrest and apoptosis.

## Results

### Vpr Induces Cell Cycle Arrest in G2 Phase and Apoptosis in HeLa/Fucci2 Cells

**Figure 1:**
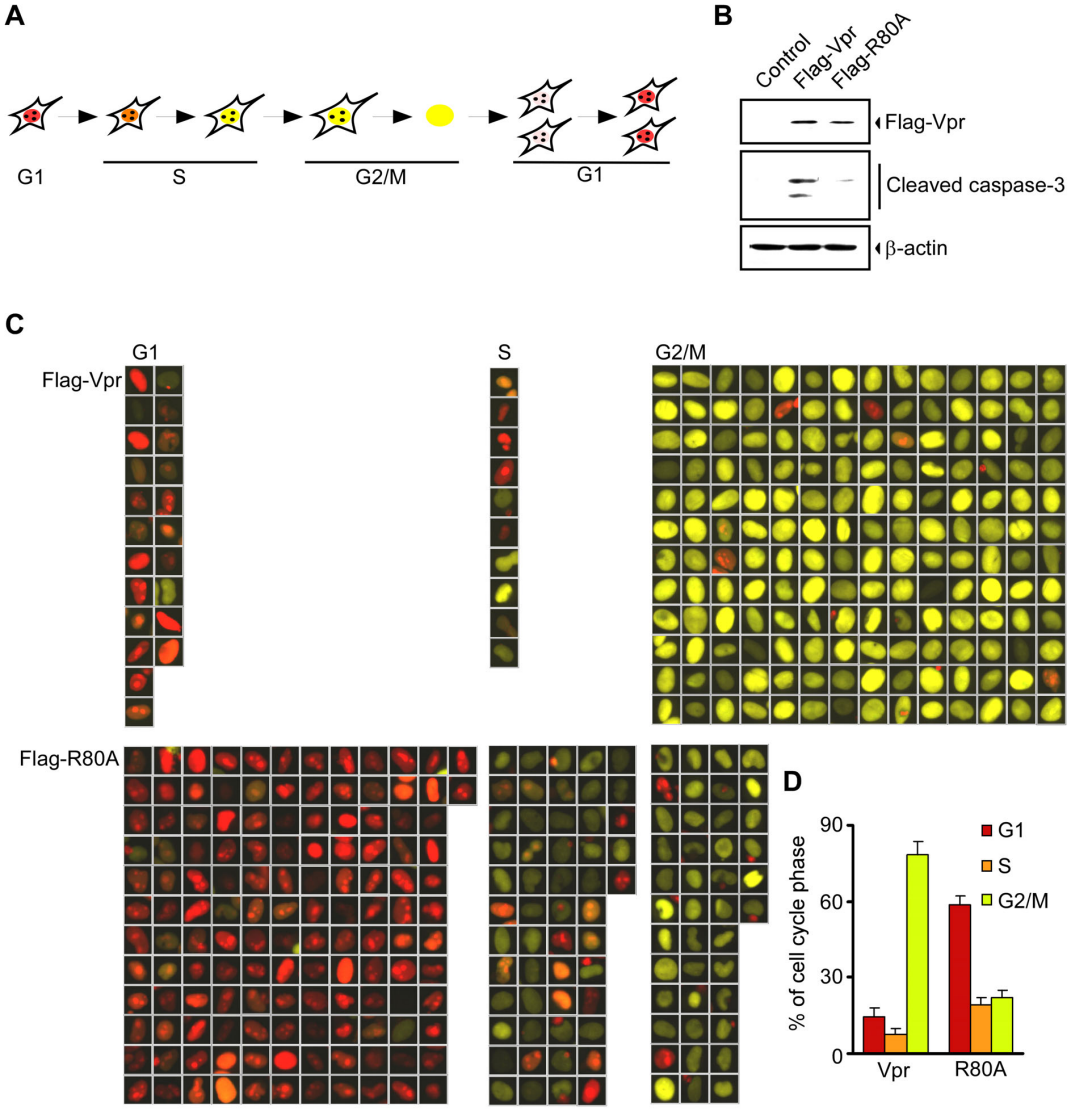
Vpr induces cell cycle arrest in G2 phase and apoptosis in HeLa/Fucci2 cells. (A) Schema illustrating the cell cycle alteration(s) observed in HeLa/Fucci2 cells. HeLa/Fucci2 cells exhibit red nuclei in G1 and yellow nuclei in S/G2/M phase. (B) Western blot analysis for the detection of Vpr expression and apoptosis. HeLa/Fucci2 cells were transfected with pME18Neo/Flag-Vpr, pME18Neo/Flag- R80A, or pME18Neo. At 72 h after transfection, the cells were lysed and subjected to Western blot analysis with anti-Flag monoclonal antibody (MAb) M2, anti-β-actin MAb, and anti-cleaved caspase-3 (Asp175) antibody. (C) Representative images of Fucci2 fluorescence of cells in G1, S, and G2/M phases of the cell cycle from at least 200 Alexa680-positive cells that were selected automatically using CELAVIEW microscope at 72 h after transfection. HeLa/Fucci2 cells were transfected with either pME18Neo/Flag-Vpr or pME18Neo/Flag-R80A. At 72 h after transfecti on, cells were stained with anti-Flag MAb M2, followed by Alexa680 conjugated anti-mouse IgG MAb for the detection of cells expressing wild-type or R80A Vpr, and then stained with Hoechst 33342 to measure DNA content. The DNA content of Alexa680-positive cells was analyzed by using a CELAVIEW microscope. (D) Quantitation of (C). The percentages of cells are in the G1, S, and G2/M phase were indicated in the *bar graph.* Data are present as means ± SD of triplicate wells.

To visualize the dynamics of cell cycle arrest and apoptosis by Vpr, the temporal patterns of cell cycle regulation following Vpr expression were monitored in HeLa/Fucci2 cells [33]. The HeLa/Fucci2 cell system was chosen because it allows dual-color imaging, in which the nuclei emit red fluorescence during G1 phase and yellow fluorescence in other phases (S, G2 or M) as they oscillate through the cell cycle (Figure 1A).

To examine whether Vpr induces cell cycle arrest at the G2 phase and promotes apoptosis in HeLa/Fucci2 cells, the cells were transfected with the pME18Neo expression vector encoding Flag-tagged wild-type Vpr (Flag-Vpr) or mutant R80A (Flag-R80A), which is defective in the induction of G2 arrest and apoptosis [37]. At 72 h post-transfection, the expression of Vpr protein was assessed by Western blot analysis of cell extracts using the monoclonal antibody (MAb) M2, which recognizes the Flag tag (Figure 1B). Single bands with an apparent molecular mass consistent with the predicted sequences of Flag-Vpr or mutant Flag-R80A were observed. The active form of caspase-3, which plays an important role in apoptosis, was detected in Flag-Vpr-expressing cells but not in negative control Flag-R80A-expressing cells by Western blot analysis with anti-activated caspase-3 (Asp175) antibody, demonstrating that apoptosis was induced in Vpr-expressing HeLa/Fucci2 cells. Seventy-two hours after transfection, cells were fixed, permeabilized and stained with anti-Flag MAb M2 for the detection of Vpr expression and with Hoechst 33342 for the measurement of DNA content. The DNA content of individual cells was quantified and the cell cycle profiles were obtained using a CELAVIEW microscope, which can analyze DNA content in a large number of cells as effectively as flow cytometry, in addition to monitoring and acquiring cell images. As shown in Figure 1C and D, CELAVIEW analysis revealed that in cells transfected with the Flag-Vpr vector were mainly in G2 phase (approximately 78.3%), whereas cells transfected with the control Flag-R80A vector were not (approximately 22.1%). Indeed, most Vpr-expressing HeLa/Fucci2 cells exhibited yellow nuclei, strongly indicating that G2 cell cycle arrest was induced in these cells.

### Visualizing the Temporal Dynamics of the Induction of Cell Cycle Arrest and Apoptosis by Vpr

To identify Vpr-expressing HeLa/Fucci2 cells during live-cell imaging, we constructed a co-expression vector that contained Flag-Vpr, an internal ribosomal entry site (IRES), and ECFP. The vector was expressed in HeLa cells, and Vpr-expressing cells were stained with anti-Flag MAb M2 followed by an Alexa594 secondary antibody at 24 h post-transfection. As shown in Figure S1, Flag-Vpr (red) was localized predominantly to the nucleus and nuclear membrane, and ECFP (blue) was localized to both the nucleus and cytoplasm. Single bands with apparent molecular masses consistent with the predicted sequences of Flag-Vpr and ECFP were observed by Western blot analysis (Figure S2). Thus, all Vpr-expressing cells were ECFP-positive, indicating that ECFP was a useful marker for the detection of cells that expressed Vpr.

**Figure 2:**
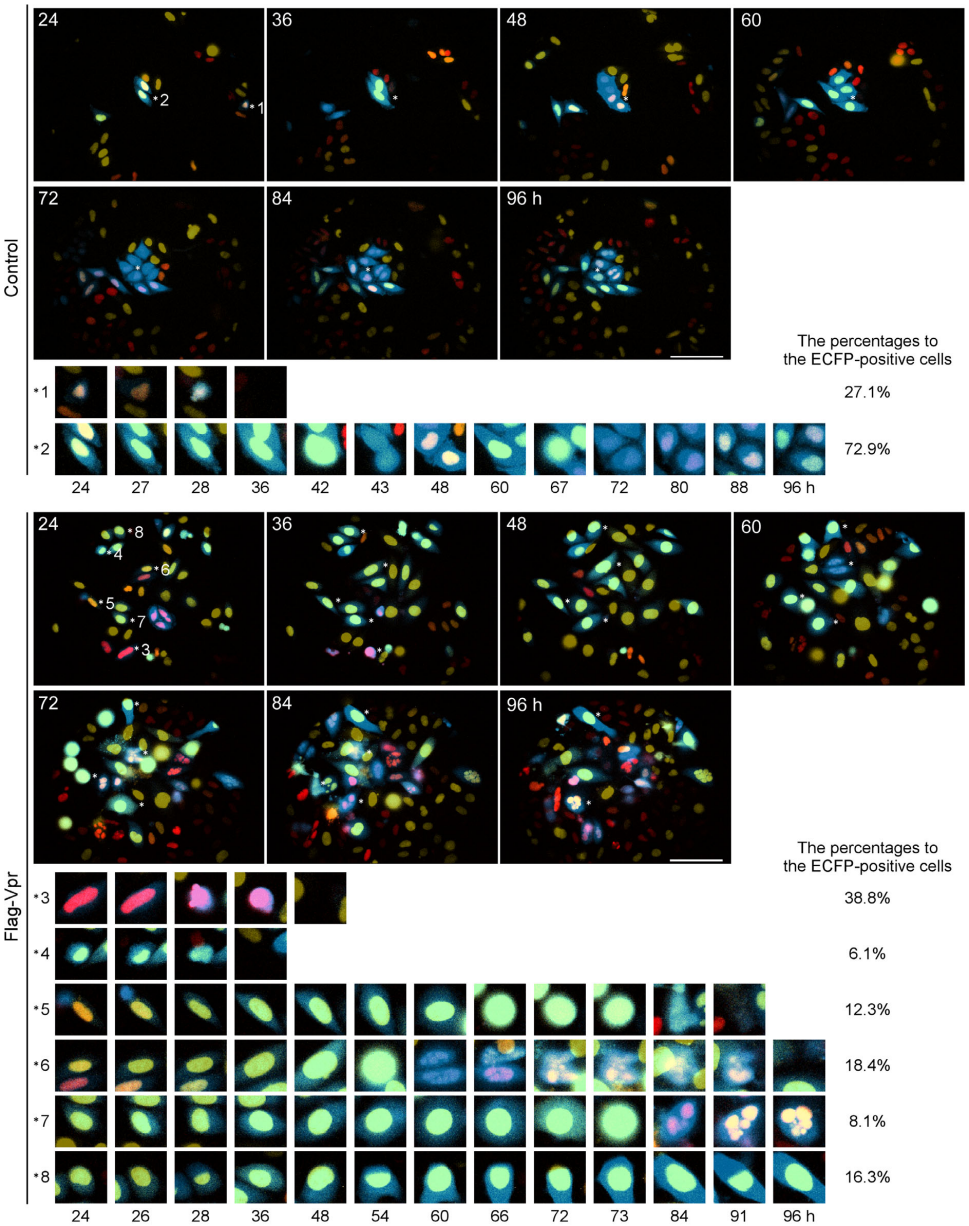
Visualizing Vpr-induced cell cycle arrest and cell death. HeLa/Fucci2 cells were transfected with pME18Neo/Flag-Vpr-IRES-ECFP or the control pME18Neo/Flag-IRES-ECFP. At 24 h after transfection, cells were monitored by time-lapse imaging using LCV110 Imaging System at 15 minute intervals until 96 h post-transfection. Cells showing red or yellow fluorescence in the nucleus are in the G1 or S/G2/M phases of the cell cycle, respectively. ECFP and Fucci2 fluorescence is shown at 24, 36, 48, 60, 72, 84, and 96 h post-transfection. Eight ECFP-positive cells were selected as representative images, and are marked as *1 to *8 on the right of each cell. The scale bars represent 100 μ. High-magnification images of ECFP-expressing cells *1 to *8 are shown for cells transfected with the control pME18Neo/Flag-IRES-ECFP (*1 and *2) at 24, 27, 28, 36, 42, 43, 48, 60, 67, 72, 80, 88, or 96 h, or pME18Neo/Flag-Vpr-IRES-ECFP (*3 to *8) at 24, 26, 28, 36, 48, 54, 60, 66, 72, 73, 84, 91, or 96 h after transfection.

HeLa/Fucci2 cells were transfected with either pME18Neo/Flag-Vpr-IRES-ECFP or the control pME18Neo/Flag-IRES-ECFP, and time-lapse images were monitored using an Olympus LCV110 Imaging System from 24 h to 96 h after transfection (Figure 2, Video S1 and S2). Although approximately 27.1% of the ECFP-positive cells transfected with the control vector exhibited red or yellow nuclei and underwent cell death during observation (*1 in Figure 2), approximately 72.9% exhibited cell division from 42 h to 43 h and red-to-yellow nuclear color conversion from 80 h to 96 h, indicating that the cells were progressing normally through the cell cycle (*2 in Figure 2). The lengths of the G1 and S/G2/M phases were about 12 h and 10 h, respectively. Indeed, the percentage of ECFP-positive cells was not greatly decreased in the control cells (Figure S3). By contrast, there was a notable reduction in the percentage of ECFP-positive, Flag-Vpr-expressing cells (Figure S3). Interestingly, 38.8% of the cells in G1 phase with red nuclei, and 6.1% of the cells in G2 phase with yellow nuclei, died within 48 h after transfection, as shown in *3 and *4 in Figure 2. On the other hand, approximately 55.1% of these cells underwent cell cycle arrest in G2 phase with yellow nuclei, and G2 phase was prolonged for at least 24 h. After cell cycle arrest in G2 phase, we also observed long-term mitotic cell rounding with yellow nuclei, as shown in *5 to *8 in Figure 2. Approximately 12.3% of these cells underwent cell death in M phase (*5 in Figure 2), and approximately 18.4% underwent micronuclei formation after abnormal mitosis, followed by cell death in G1 phase (*6 in Figure 2). In approximately 8.1% of the cells, the nuclei changed color from yellow to red without the cells undergoing cell division, indicating “nuclear mis-segregation” (*7 in Figure 2). This process is characterized by nuclear envelope breakdown and subsequent chromosome fragmentation, both of which are a consequence of failure in mitosis [38]. In 16.3% of the cells, the cell cycle was arrested throughout the observation period (*8 in Figure 2). Interestingly, time-lapse imaging of the HeLa/Fucci2 cells showed that although Vpr arrested the cell cycle at G2 phase, it hardly induced cell death in S or G2 phase.

### Visualization of the Temporal Dynamics of Cell Cycle Arrest and Apoptosis Following Infection with an Adenovirus Expressing Vpr

**Figure 3:**
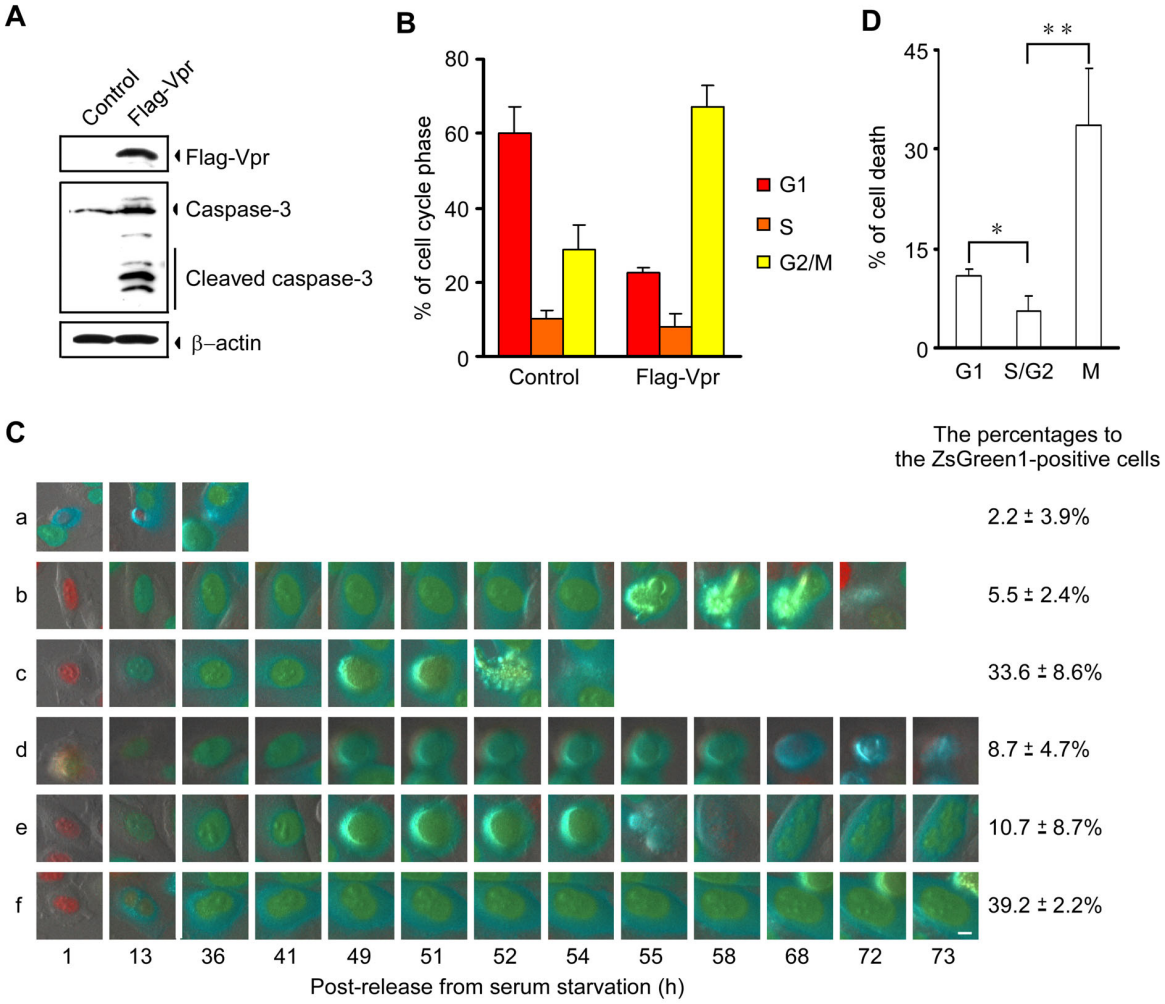
G2 arrest and cell death following adenoviral expression of Vpr. (A and B) HeLa cells were infected with adenoviral vector pAdeno-X/Flag-Vpr-IRES-ZsGreen1 or the control vector pAdeno-X/IRES-ZsGreen1 at MOI 100 and cultured for 72 h. (A) Cells were lysed and subjected to Western blot analysis with anti-Flag MAb, anti-caspase-3 polyclonal antibody, and anti-β-actin MAb. (B) Cells were fixed and stained in 3.6% formaldehyde containing Hoechst 33342. The DNA contents of ZsGreen1-positive cells were observed under a CELAVIEW microscope. Data points are means ± SD of triplicate samples. (C and D) Serum-starved HeLa/Fucci2 cells were infected with the adenoviral vector pAdeno-X/Flag-Vpr-IRES-ZsGreen1 at MOI 50 in DMEM containing 0.3% FCS. (C) At 23 h post-infection, cells were washed to remove uninfected virus and further cultured for 1 h in DMEM containing 10% FCS. Then, the cells were monitored by time-lapse imaging using the LCV110 Imaging System at 15 minute intervals at 73 h after release from serum starvation. Cells showing the cyan fluorescence of ZsGreen1 in entire cells established that the cells had been infected with adenoviral vector. Red or yellow fluorescence in nuclei show the cells are in G1 or S/G2/M phases of the cell cycle, respectively. High-magnification images of six cells (a to f) infected with the adenoviral vector are shown. The scale bar represents 5 µm. The percentages of each cell profile (a to f) of the ZsGreen1-expressing cells were estimated from triplicate dishes. At least 30 ZsGreen1-expressing cells were observed in every dish. Data are presented as means ± SD of triplicate dishes. (D) The dead cells were classified into G1, S and G2/M phases according to the color of the cell nucleus and cell morphology during observation. Data are presented as means ± SD of triplicate dishes. The asterisk indicates a statistically significant difference (*p<0.05; **p<0.01).

To confirm the temporal relationship between Vpr-induced G2 arrest and cell death, we used the adenovirus infection system and synchronized cells after release from serum starvation. We first constructed a co-expression adenoviral vector (pAdeno-X/Flag-Vpr-IRES-ZsGreen1) that expressed Flag-Vpr, an IRES, and ZsGreen1 fluorescent protein, or the control pAdeno-X/IRES-ZsGreen1 according to the manufacturer`s protocol for the Adeno-X™ expression system (Clontech Laboratories, Mountain View, CA). HeLa cells were infected with a pAdeno-X/Flag-Vpr-IRES-ZsGreen1 or the control vector pAdeno-X/IRES-ZsGreen1 at a multiplicity of infection (MOI) of 100. At 72 h post-infection, the active form of caspase-3 was detected in Flag-Vpr-expressing cells but not in control cells by Western blot analysis with anti-caspase-3 antibody (Figure 3A), and G2 arrest was observed in Flag-Vpr expressing cells using a CELAVIEW microscope (Figure 3B). Next, to synchronize the cell cycle, we used serum starvation. When HeLa/Fucci2 cells were cultured in DMEM containing 0.3% fetal bovine serum (FBS), the percentage of cells in G0/G1 phase with red nuclei was higher than in cells cultured in DMEM containing 10% FBS (approximately 76.5% in serum-starved cells *vs.* 64.5% in non-serum-starved cells) (data not shown).

We monitored the nuclear color of serum-starved HeLa/Fucci2 cells infected with the adenoviral vector pAdeno-X/Flag-Vpr-IRES-ZsGreen1 at MOI 50 in DMEM containing 0.3% FBS. At 23 h post-infection, we changed the medium to DMEM containing 10% FBS and cultured the cells for an additional 1 h. Live-cell imaging using LCV110 at this point revealed that most cells were mainly in G0/G1 phase with red nuclei and did not express ZsGreen1. At 36 h after release from serum starvation, ZsGreen1 fluorescence (cyan) was seen in most of the cells, indicating that infection had been established. In approximately 2.2% of the cells in G1 phase, cell death was observed up to 36 h after release from serum starvation (“a” in Figure 3C and “G1” in 3D; corresponding to *3 of Figure 2). Other cells underwent cell cycle arrest at G2 phase with yellow nuclei (“b to f” in Figure 3C). After cell cycle arrest, approximately 5.5% of the cells underwent cell death in S/G2/M phase without long-term mitotic cell rounding (“b” in Figure 3C and “S/G2” in 3D; corresponding to *4 of Figure 2). On the other hand, approximately 33.6% of the cells entered M phase and exhibited long-term mitotic cell rounding before cell death (“c” in Figure 3C and “M” in 3D; corresponding to *5 of Figure 2). After rounding, approximately 8.7% of the cells underwent abnormal cell division and subsequent cell death at G1 phase (“d” in Figure 3C and ”G1” in 3D; corresponding to *6 of Figure 2). Approximately 10.7% of the cells did not undergo cell death but exhibited nuclear mis-segregation, and progressed through the cell cycle with micronuclei (“e” in Figure 3C; corresponding to *7 of Figure 2). Approximately 39.2% of the cells did not undergo cell death and remained in G2 phase or exhibited long-term mitotic cell rounding (“f” in Figure 3C; corresponding to *8 of Figure 2). As shown in Figure 3D, only 5.5% of the cells exhibited cell death at S and G2 phase. Thus, although the ratios of the populations shown in a to f in Figure 3C were different from those of *3 to *8 in Figure 2, these results, which were obtained using the adenovirus infection system and synchronized HeLa/Fucci2 cells, confirm the results obtained with HeLa/Fucci2 cells transiently transfected with the Vpr expression vector, i.e., that Vpr arrests the cell cycle in G2 phase, but does not induce cell death in S or G2 phase.

### Visualizing Vpr-induced Apoptosis Accompanied by Caspase-3 Activation

To determine whether Vpr induces cell death via caspase-3 dependent apoptosis, we used SCAT3.1 [36], which is a FRET-based indicator of caspase-3 activation. SCAT 3.1 is composed of ECFP, a caspase-3-cleaved linker, and the enhanced yellow fluorescence protein Venus. SCAT3.1 enables the monitoring of caspase-3 activation in single living cells by measuring the change in fluorescence that accompanies FRET disruption.

We first constructed pME18Neo/Flag-Vpr-IRES-SCAT3.1, which contained Flag-Vpr, an IRES, and SCAT3.1. After transfection with this vector or with the control vector pME18Neo/Flag-IRES-SCAT3.1, HeLa cells were stained with anti-Flag MAb M2 followed by an Alexa594 secondary antibody. As shown in Figure S4, at 24 h post-transfection, Flag-Vpr (red) was localized in the nucleus and nuclear membrane, and ECFP (blue) and Venus (yellow) of SCAT3.1 were localized in the cytoplasm, since SCAT3.1 contains nuclear export signals (NES) at its C- and N-termini to distinguish the yellow and cyan fluorescence of SCAT3.1 from the yellow fluorescence of mVenus-hGeminin (1/110) and red fluorescence of mCherry-hCdt1 (30/120) in the nuclei of HeLa/Fucci2 cells. We examined the expression of Flag-Vpr and SCAT3.1 in HeLa/Fucci2 cells transfected with either pME18Neo/Flag-Vpr-IRES-SCAT3.1 or pME18Neo/Flag-IRES-SCAT3.1 as a control by Western blot analysis. We detected the expression of Flag-Vpr and full-length and cleaved SCAT3.1 in Flag-Vpr-expressing cells at 24 h post-transfection (Figure S5), indicating that Vpr induces apoptosis through the activation of caspase-3 in HeLa/Fucci2 cells.

**Figure 4:**
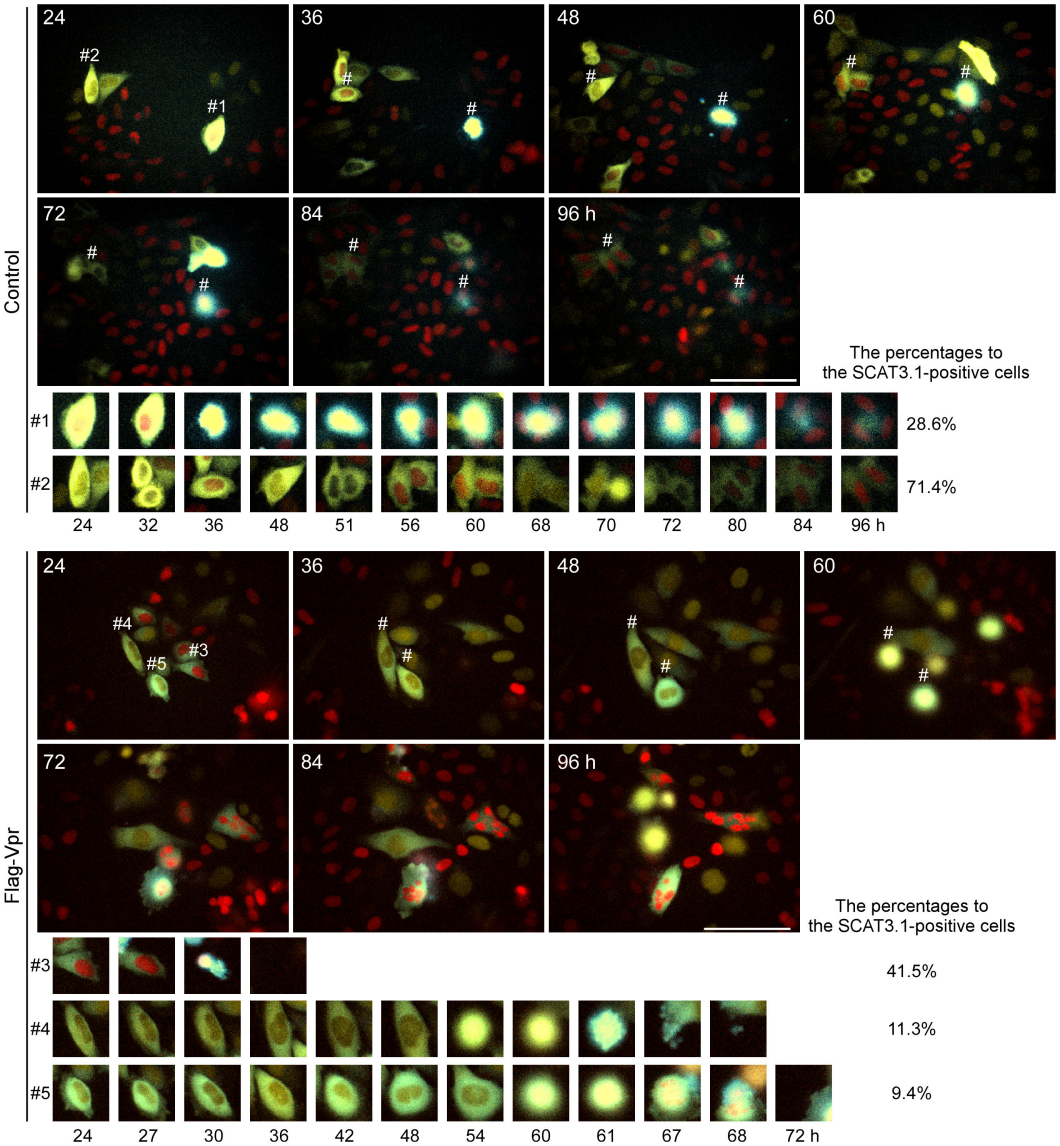
Vpr induces cleavage of SCAT3.1 at the same time as caspase-3 activation. HeLa/Fucci2 cells were transfected with pME18Neo/Flag-Vpr-IRES-SCAT3.1 or the control pME18Neo/Flag-IRES-SCAT3.1. At 24 h after transfection, cells expressing SCAT3.1 in the cytoplasm were observed with the LCV110 Imaging System at 15 minute intervals for 96 h. Nuclei with red fluorescence are in G1 phase. Yellow or cyan fluorescence in the cytoplasm identifies inactivated or activated caspase-3, respectively. The ECFP and Venus fluorescence of SCAT3.1 and the mCherry and Venus fluorescence of Fucci2 are shown at 24, 36, 48, 60, 72, 84, and 96 h post-transfection. Five SCAT3.1-positive cells were selected as representative images and are marked as #1 to #5 in the upper part of each cell. The scale bars represent 100 µh. High-magnification images of SCAT3.1-expressing cells #1 to #5 are shown for cells transfected with the control pME18Neo/Flag-IRES-SCAT3.1 (#1 and #2) at 24, 32, 36, 48, 51, 56, 60, 68, 70, 72, 80, 84, or 96 h after transfection, or pME18Neo/Flag-Vpr-IRES-SCAT3.1 (#3 to #5) at 24, 27, 30, 36, 42, 48, 54, 60, 61, 67, 68, or 72 h after transfection.

We performed time-lapse imaging of HeLa/Fucci2 cells transfected with pME18Neo/Flag-Vpr-IRES-SCAT3.1 (total cell number; 53) or pME18Neo/Flag-IRES-SCAT3.1 (total cell number; 28) as a control until 96 h post-transfection (Figure 4, Video S3 and S4). A minority of HeLa/Fucci2 cells (approximately 28.6%) transfected with control vector showed SCAT3.1 cyan fluorescence, indicative of caspase-3 activation, in the cytoplasm. Caspase activation spread to the nucleus and was followed by cell death (see cell #1 in Figure 4). Yellow SCAT3.1 fluorescence, indicative of inactive caspase-3, was observed in the cytoplasm of most cells (approximately 71.4%) transfected with the control vector (#2 in Figure 4), indicating that these cells were proliferating normally. Interestingly, cell death accompanied by a change in the fluorescence of SCAT3.1 from yellow to cyan, indicating caspase-3 activation, was observed more frequently in Vpr-expressing cells over a range of time points (Figure 4) than in cells transfected with the control vector. For example, 41.5% of Vpr-expressing cells were in G1 phase with red nuclei and induced caspase-3 activation during apoptosis, as shown in #3 in Figure 4 (corresponding to *3 of Figure 2). In addition, compared to the #2 cell, the 530/480 nm emission ratios were notably decreased in the #3 cell at 30 h post-transfection indicating that apoptosis occurred in the #3 cell in a caspase-3-dependent manner (Figure 4 and S6). Furthermore, Vpr-expressing HeLa/Fucci2 cells that exhibited active caspase-3 in M phase (#4 in Figure 4, approximately 11.3%; corresponds to *5 in Figure 2) and in G1 phase (#5 in Figure 4; approximately 9.4%; correspond to *6 in Figure 2) underwent apoptosis after G2 cell cycle arrest. These results suggest that, at every time point, Vpr induces apoptosis in a caspase-3-dependent manner.

### Characterization of G2 Arrest and Apoptosis by Shield1-responsive DD Fusion Vpr in HeLa Cells using Reversible Destabilization

To examine the reversibility of Vpr effects on G2 cell cycle arrest and apoptosis, we used a ProteoTuner system for rapid destabilization and stabilization of Vpr [39]. When expressed in mammalian cells, the DD is constitutively degraded in a proteasome-dependent manner, including any other protein fused to the DD. However, a small (750 Da), membrane permeable, stabilizing ligand, Shield1, can prevent DD-tagged proteins from being degraded, which allows the protein to accumulate in the cells [39]. Therefore, the DD-tag would enable Vpr to be stabilized and destabilized speedily by Shield1.

**Figure 5:**
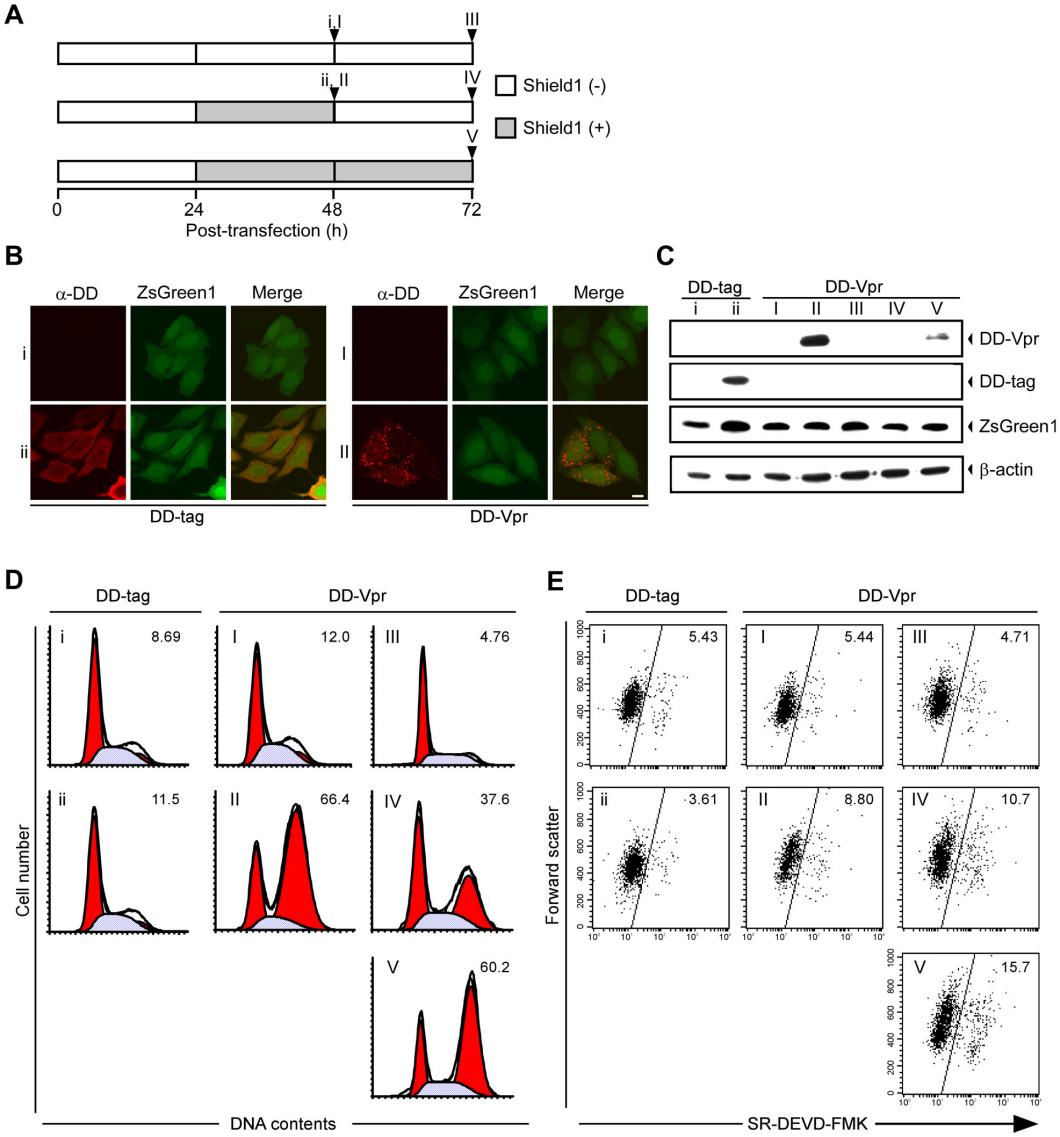
Cell cycle arrest and cell death in cells expressing a destabilizing domain (DD) Vpr fusion in the presence and absence of Shield1. (A) Schemas illustrating the treatment with Shield1. HeLa cells were transfected with pME18Neo/DD-Vpr-IRES-ZsGreen1 (I-V) or the control pME18Neo/DD-IRES-ZsGreen1 (i and ii) and cultured for 24 h. The cells were then treated in the absence (i and I) or presence (ii and II) of 500 nM Shield1 for 24 h. The cells were washed to remove Shield1 and further cultured in the absence (IV) or presence (V) of 500 nM Shield1 for 24 h. (B) The cells were fixed, permeabilized, stained with anti-DD MAb followed by Alexa594 secondary MAb, and analyzed by confocal laser scanning microscopy. Cells showing red and green fluorescence express DD-Vpr and ZsGreen1, respectively. The scale bar represents 10 µm. (C) Cells were lysed and subjected to Western blot analysis with anti-DD MAb, anti-ZsGreen1 antibody, and anti-β-actin MAb. (D) Cells were stained with PI. ZsGreen1-positive cells were analyzed by flow cytometry using CELL Quest for acquisition and ModFit LT for quantitative analysis of DNA content. The percentage of cells in G2/M phase is indicated in the upper right of each graph. (E) Cells were stained with SR-DEVD-FMK to identify cells with active caspase-3. The percentage of cells with active caspase-3 was measured by flow cytometry in ZsGreen1-positive cells.

To examine the effect of the DD-tag on stabilization, localization, G2 arrest and apoptosis by Vpr, we constructed a DD-tagged Vpr expression vector pME18Neo/DD-Vpr-IRES-ZsGreen1 and a negative control, pME18Neo/DD-IRES-ZsGreen1, and transfected these constructs into HeLa cells. At 24 h post-transfection, cells were treated with 500 nM Shield1 for 24 h. Shield1 was then washed away with medium and further cultured in the presence or absence of 500 nM Shield1 for 24 h (Figure 5A). The subcellular localization of DD-Vpr was analyzed. As shown in Figure 5B (ii and II), DD mainly localized to the cytoplasm, while DD-Vpr had a nuclear localization with punctate, cytoplasmic staining in HeLa cells at 24 h after treatment with Shield1. In contrast, DD and DD-Vpr expression was not detected in the absence of Shield1 (Figure 5B i and I). All DD-Vpr-expressing cells were ZsGreen1-positive, indicating that ZsGreen1 is a useful marker for the detection of cells expressing Vpr. Western blot analysis using anti-DD MAb detected DD-Vpr expression at 24 h (Figure 5C lane II) and 48 h (Figure 5C lane V) after Shield1 addition, while it detected rapid degradation of DD-Vpr after Shield1 removal (Figure 5C lane IV). By contrast, no specific band was detected in the absence of Shield1 (Figure 5C lanes I and III). Correspondingly, although DD-Vpr induced G2 arrest in the presence of Shield1 (approximately 66.4% and 60.2% of ZsGreen1-positive cells were in G2/M at 24 h and 48 h after Shield1 addition, respectively), this effect was decreased by the rapid degradation of DD-Vpr caused by Shield1 removal (approximately 37.6% of ZsGreen1-positive were in G2/M) (Figure 5D). These results clearly demonstrate the partial reversibility of the effect of DD-Vpr on G2 cell cycle arrest functions in HeLa cells.

DD-Vpr-expressing cells induced apoptosis more effectively in the presence of Shield1 (approximately 15.7% of ZsGreen1-positive cells were apoptotic at 48 h after Shield1 addition) than in the absence of Shield1 (approximately 4.71% ZsGreen1-positive cells). Moreover, the apoptotic cell number was maintained at 24 h after Shield1 removal (approximately 10.7% of ZsGreen1-positive cells) (Figure 5E). These results suggest that the expression of DD-Vpr for 24 h does not induce apoptosis as effectively as its constitutive expression in HeLa cells.

### Visualizing the Regulation of G2 Arrest and Apoptosis by Shield1-responsive DD Fusion Vpr

**Figure 6:**
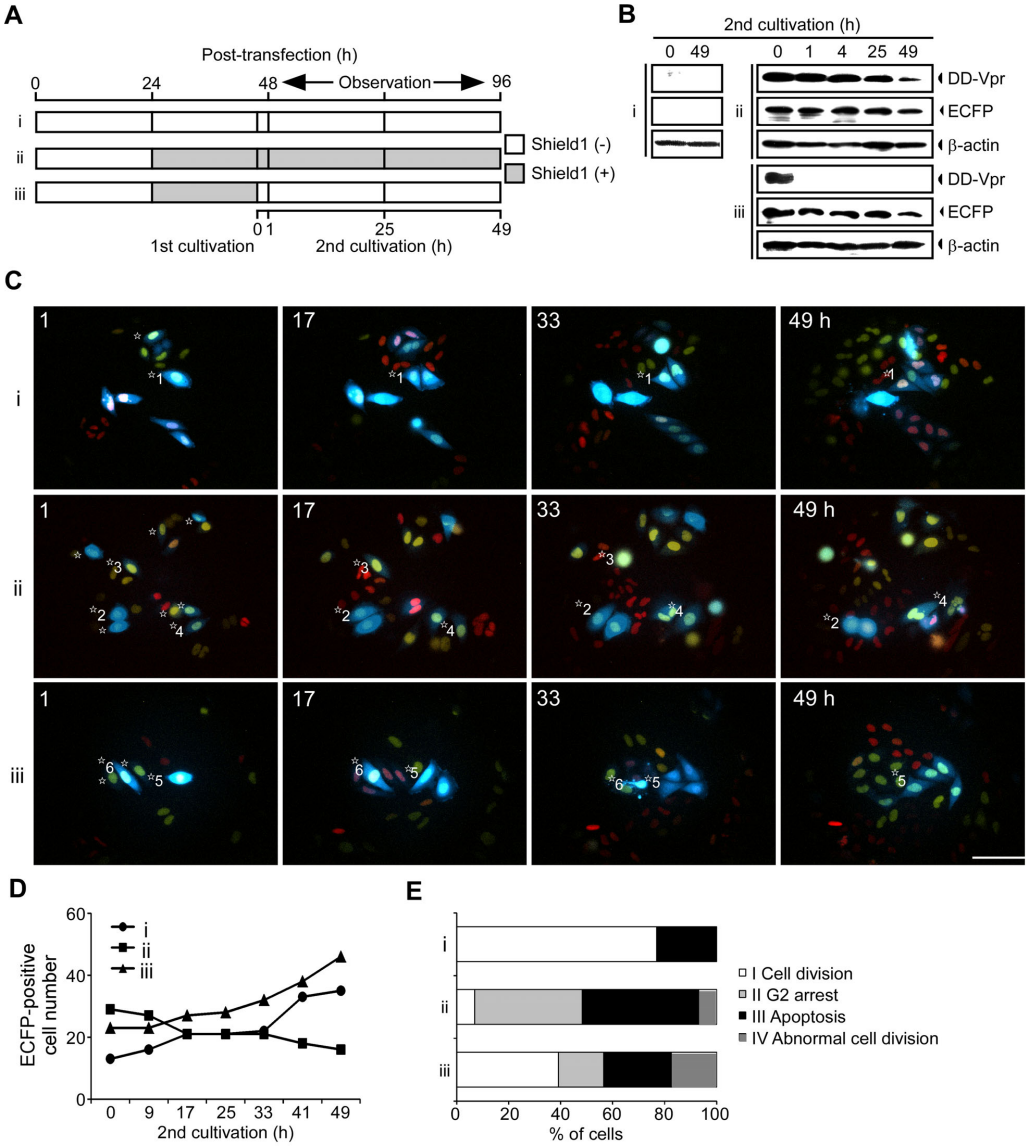
Expression of DD-Vpr is required for persistent G2 arrest and apoptosis after G2 arrest. (A) Schemas illustrating the treatment with Shield1. HeLa/Fucci2 cells were transfected with pME18Neo/DD-Vpr-IRES-ECFP. Twenty-four hours after transfection, the cells were cultured in the absence (i) or presence (ii and iii) of 500 nM Shield1 for 23 h (1st cultivation). The cells were washed to remove Shield1 and further cultured in the presence (ii) or absence (iii) of 500 nM Shield1 for 49 h (2nd cultivation). (B) DD-Vpr expression was monitored using Western blot analysis with anti-DD MAb, anti-GFP MAb, and anti-β-actin MAb at 0, 1, 4, 25, and 49 h after the 2nd cultivation. (C-E) ECFP-expressing cells that were in G2 phase 1 h after the 2nd cultivation (marked as ^☆^ on the left of each cell) were selected and monitored by time-lapse imaging using LCV110 Imaging System at 15 minute intervals for a further 48 h. (C) Cells showing red or yellow fluorescence in the nucleus are in G1 or S/G2/M phase, respectively. ECFP and Fucci2 fluorescence are shown at 1, 17, 41, and 49 h after the 2nd cultivation. Six ECFP-positive cells that were in G2 phase at 1 h after the 2nd cultivation were selected as representative images and are marked as ^☆^1 to ^☆^6 on the left of each cell. The scale bars represent 100 µm. (D) The total number of ECFP-expressing cells that had been in G2 phase at 1 h after the 2nd cultivation were counted in particular areas. (E) The effect of continuous DD-Vpr expression in G2 arrested cells was determined by assessing Fucci2 fluorescence and morphology. The proportion of cells undergoing normal cell division (white), persistent G2 arrest (gray), cell death (black), and abnormal cell division (horizontal line) were calculated and plotted.

We performed live-cell imaging to analyze the effect of DD-Vpr on G2 cell cycle arrest and apoptosis functions using HeLa/Fucci2 cells transfected with pME18Neo/DD-Vpr-IRES-ECFP. Twenty-four hours after transfection, cells were cultured with 500 nM Shield1 for 23 h (1st cultivation). Shield1 was then washed away and the cells were then cultured in the presence or absence of 500 nM Shield1 (2nd cultivation) (Figure 6A). Western blot analysis clearly demonstrated that the expression of DD-Vpr continued until 49 h after Shield1 addition (2nd cultivation), and that DD-Vpr degradation started 1 h after the removal of Shield1 (Figure 6B). DD-Vpr expression was not detected when Shield 1 was absent during the final 23 h of the 1st cultivation and the final 49 h of the 2nd cultivation.

We sought to explore the effect of transient expression of DD-Vpr on apoptosis after G2 arrest. At 1 h after the 2nd cultivation, we selected ECFP-expressing cells in G2 phase with yellow nuclei (^☆^of Figure 6C, panel i–iii) and monitored the temporal dynamics of cell cycle progression of these cells for 48 h using an Olympus LCV110 Imaging System (Figure 6C and Video S5–7). In the absence of Shield1, the ECFP-positive cells in G2 phase proliferated normally through cell cycle for 48 h (^☆^1 of Figure 6C, panel i). Indeed, the cell number slowly increased during the 48 h culture (Figure 6D). In contrast, in the presence of Shield1, the number of ECFP-expressing cells slowly decreased (Figure 6D). Time-lapse imaging using HeLa/Fucci2 cells clearly demonstrated that ECFP-expressing cells cultured in the presence of Shield1 could be classified into four types: type I cells, which divided and had a normal cell cycle progression; type II cells, which underwent G2 cell cycle arrest throughout the observation period (^☆^2 of Figure 6C, label ii); type III cells, which underwent apoptosis (^☆^3 of Figure 6C, label ii); and type IV cells, which displayed evidence of abnormal cell division (^☆^4 of Figure 6C, label ii). The ECFP-expressing cells that had been in G2 phase at 1 h after the addition of Shield1 in the 2nd cultivation were approximately 6.9% type I, 41.4% type II, 44.8% type III, and 6.9% type IV cells. Interestingly, in the ECFP-expressing cells, the proportion of type I cells was considerably higher (approximately 39.1% of the ECFP-expressing cells; ^☆^5 of Figure 6C, label iii) after the removal of Shield1 than in cells grow in the presence of Shield1. Moreover, the removal of Shield1 resulted in a dramatic reduction in the population of type II cells, which were arrested at the G2 phase of cell cycle (approximately 17.4% of the ECFP-expressing cells). By contrast, the size of the type III cell population, which underwent apoptosis (approximately 26.1% of the ECFP-expressing cells; ^☆^6 of Figure 6C, label iii) after the removal of Shield1 was similar to that of the type III cell population in the absence of Shield1 (approximately 26.1 *vs* 23.1% of the ECFP-expressing cells; ^☆^6 of Figure 6C, label i). Thus, by using time-lapse imaging, we could observe the dynamics of morphological changes and the reversibility of Vpr effects on G2 arrest and subsequent apoptosis.

## Discussion

Previous studies have indicated that Vpr regulates cell cycle progression and apoptosis both positively and negatively; however, the molecular mechanism(s) underlying the regulation of the two processes by Vpr remain obscure [12]–[14], [19], [23], [26]–[31]. To understand the biological events affected by Vpr, particularly the regulation of cell cycle arrest and apoptosis, this study used four new methods: (i) cell cycle profiling using a CELAVIEW microscope for the analysis of DNA content in large numbers of cells, (ii) time-lapse imaging of HeLa/Fucci2 cells for the monitoring of the dynamics of Vpr-induced cell cycle arrest, (iii) functional live imaging of caspase-3 activation with SCAT3.1 during Vpr-induced apoptosis, and (iv) Shield1-responsive DD fusion Vpr for the examination of the reversibility of Vpr functions. Three major conclusions can be drawn from the data. First, time-lapse imaging of HeLa/Fucci2 cells and CELAVIEW imaging of HeLa/Fucci2 cells showed that, although Vpr arrested the cell cycle at G2 phase, Vpr-induced apoptosis did not occur in S or G2 phase. Thus, this finding, which is similar to the results obtained by live imaging of synchronized HeLa/Fucci2 cells infected with an adenoviral vector expressing Vpr, confirms that Vpr arrests the cell cycle in G2 phase, but does not induce cell death in S or G2 phase. Second, by performing time-lapse imaging of HeLa/Fucci2 cells in combination with SCAT3.1, we clearly demonstrated that caspase-3 activation was required for Vpr-induced apoptosis. Interestingly, caspase-3 activation did not occur just after G2 arrest, but was induced in M phase and G1 phase after G2 cell cycle arrest. Third, time-lapse imaging using a Shield1-responsive DD fusion showed that G2 cell cycle arrest and subsequent apoptosis induced by Vpr is reversible. In addition, transient expression of Vpr for about 1 day was insufficient to maintain continuous G2 arrest and apoptosis after G2 arrest. Thus, the most interesting aspects of this study was the visualization of the morphological dynamics of Vpr-induced cell death and cell cycle arrest.

**Figure 7:**
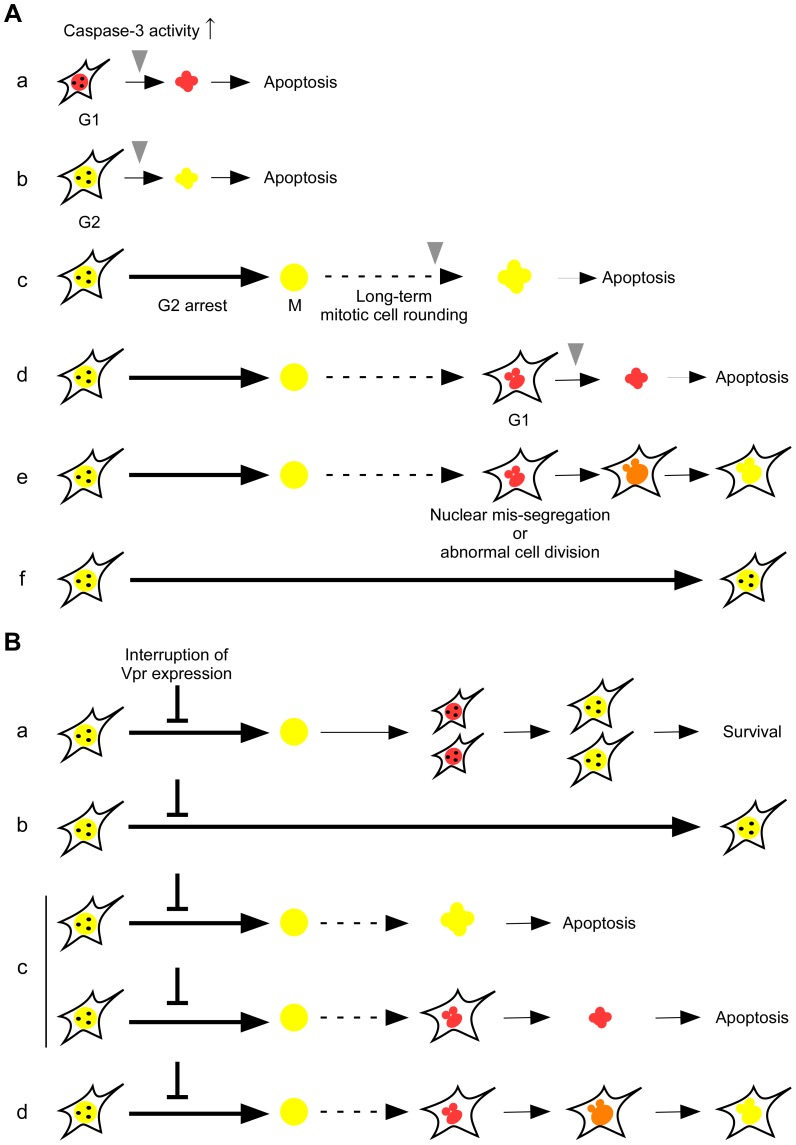
Proposed model to explain the temporal relationships between various Vpr functions. (A) Vpr induces apoptosis at G1 (a) and G2 (b) without G2 arrest. Vpr induces G2 arrest and subsequent long-term mitotic cell rounding following apoptosis (c) or nuclear mis-segregation or abnormal cell division (d and e). After nuclear mis-segregation or abnormal cell division, most of the cells undergo apoptosis (d) and other cells survive with micro nuclei (e). Furthermore, some Vpr-expressing cells underwent G2 arrest throughout the observation period (f). All apoptosis was dependent on caspase-3. Bold arrow, G2 arrest; arrow with dotted line, long-term mitotic cell rounding; and gray arrowhead, caspase-3 activation. (B) Interruption of Vpr expression leads to four phenomena: cell division with normal cell cycle progression (a), G2 arrest (b), G2 arrest with apoptosis (c), and G2 arrest with abnormal cell division (d).

A schematic representation of the relationship between Vpr-induced cell cycle arrest and apoptosis is depicted in Figure 7A. Although Vpr induced apoptosis at various time points; for example, in cells in G1 phase (a), S or G2 phase (b), and M phase (c), and in cells in G1 phase (d) with nuclear mis-segregation or abnormal cell division after long-term mitotic cell rounding, Vpr-induced apoptosis was rarely observed in S or G2 phase. This was confirmed by using synchronized HeLa/Fucci2 cells infected with an adenoviral vector expressing Vpr, which showed that Vpr induced G2 arrest in approximately 97.7% of the ZsGreen1-positive cells, but induced cell death in only 5.5% of the cells in S or G2 phase. The result suggests that Vpr causes G2 arrest, but does not induce cell death, in S or G2 phase. Thus, Vpr may exert different functions in each phase of the cell cycle, i.e. in G1 and M phase, Vpr may induce apoptosis, and in G2 phase, Vpr may induce cell cycle arrest. Interestingly, our study showed that Vpr-induced apoptosis (a–d) was caspase-3 activation dependent. A small number of cells in G1 phase survived with micro nuclei after experiencing nuclear mis-segregation or abnormal cell division and continued to progress through the cell cycle (e), while others continued to G2 arrest or engaged in long-term mitotic cell rounding (f), indicating that Vpr may regulate apoptosis, both positively and negatively. Moreover, we observed long-term mitotic cell rounding, nuclear mis-segregation and subsequent micronuclei formation after G2 arrest in Vpr-expressing cells. This is consistent with previous results showing prolonged mitosis and abnormal mitosis in Vpr-expressing cells [40], [41], and with results showing the induction of micronuclei formation and aneuploidy by Vpr [40], [42]. The data indicate that Vpr-induced aneuploidy results from events initiated by G2 cell cycle arrest.

As shown in Figure 7B, the removal of Shield1 induced a rapid degradation of DD-Vpr, which led to nearly normal cell cycle progression in cells arrested in G2 phase by Vpr (Figure 7B, a) and to a dramatic reduction in the number of cells arrested in G2 phase (Figure 7B, b), undergoing apoptosis (Figure 7B, c), and exhibiting abnormal cell division (Figure 7B, d). This result indicates that Vpr-induced G2 arrest and subsequent apoptosis may be reversible. In particular, the number of cells undergoing apoptosis after the removal of Shield1 was similar to that in the absence of Shield1, suggesting that the suppression of Vpr-induced G2 arrest may inhibit apoptosis. This result strongly supports previous data that Vpr-induced apoptosis is abrogated by the suppression of G2 arrest [26]. Furthermore, our data also showed that transient expression of Vpr for about 1 day is not sufficient to induce fully continuous G2 arrest and apoptosis in cells arrested in G2 phase (Figure 7B, a). These results indicate that Vpr-induced apoptosis after G2 arrest requires continuous expression of Vpr and a certain period of G2 arrest.

In human T cells, infection with HIV-1 induces cell cycle arrest at G2 phase. The viral proteins Vpr and Vif are independently responsible for this event [43]. On the other hand, Wang *et al*. have reported that HIV-1 Vif promotes the G1-to-S phase cell cycle transition [44], suggesting that the G1 phase is shortened by HIV-1 infection. We observed Vpr-induced apoptosis in G1 phase without G2 arrest when cells were transiently transfected with the Vpr-expression vector. Apoptosis at G1 phase may be inhibited by the possible function of Vif in efficiently induction of G2 arrest, which is significant considering the importance of G2 arrest for HIV-1 replication. Thus, Vpr and Vif may work in concert to optimize the cellular environment for effective HIV-1 infection.

Vpr associates with Cullin4 (Cul4)-based ubiquitin ligases containing DDB1 and DCAF1 to promote G2 arrest [45]–[50]. The association is observed in nuclei as chromatin-binding foci [51]. Maudet *et al*. reported that Vpr impairs cell growth through G2 arrest and G2 arrest-independent apoptosis, both of which require binding of Vpr to DCAF1 [52]. Furthermore, Huang *et al*. showed that Vpr induces apoptosis by reducing the expression of the mitochondrial membrane protein mitofusin 2 (Mfn2) via the ubiquitin ligase complex [53]. Based on the results of live imaging of HeLa/Fucci2 cells transiently transfected with the Vpr-expression vector, we propose the following mechanism: Vpr induces apoptosis at various time points; for example, in cells in G1 phase and M phase, and in cells in G1 phase with nuclear mis-segregation or abnormal cell division after long-term mitotic cell rounding. Thus, the temporal difference in the timing of Vpr-induced apoptosis would be determined by Vpr-binding proteins that are ubiquitinated and degraded by Cul4^DCAF1^ ubiquitin ligases. In the case of Vpr-induced apoptosis without G2 arrest, Mfn2 might be degraded early after the expression of Vpr via its ubiquitination. Conversely, in the case of Vpr-induced G2 arrest, Vpr would first degrade the proteins that are required for proper cell cycle progression. In the case of synchronized HeLa/Fucci2 cells infected with an adenoviral vector expressing Vpr, other mechanisms will be needed to explain why Vpr induced G2 arrest without the cells undergoing apoptosis in G2 phase in approximately 92.2% of the cells. For instance, although Vpr binds Mfn2, Vpr might not degrade Mfn2 in S and G2 phase via its ubiquitination. Clearly, it will be necessary clarify in more detail the molecular mechanism underlying the relationship between Vpr-induced apoptosis and Mfn2 degradation.

The de novo expression of Vpr induces cell cycle arrest in G2 phase and apoptosis in *vivo* [25], [54], and virion-associated Vpr induces G2 arrest and promote apoptosis without de novo expression of Vpr [55], strongly indicating that the functions of these two types of Vpr are similar. The present study showed that, although Vpr arrested the cell cycle in G2 phase, it failed to induce significant cell death in S or G2 phase. Based on the results, we propose that virion-associated Vpr induces apoptosis only when target cells are infected with HIV-1 in G1 or M phase. By contrast, when HIV-1 infects cells in S or G2 phase, virion-associated Vpr would induce G2 arrest but not apoptosis; thus, newly synthesized Vpr would contribute either additively or synergistically to G2 arrest and apoptosis after G2 arrest.

To understand the relationship between Vpr-induced G2 cell cycle arrest and apoptosis in HIV-1 infected CD4^+^ T cells from HIV-1 patients, it is important to challenge the time-lapse imaging of living primary HIV-1 infected cells. Long-term live-cell imaging using non-adherent CD4+ T cells would help define the direct and indirect effects of Vpr-mediated G2 arrest and apoptosis in primary HIV-1 infected cells, although this method is currently very challenging because of microscope focusing issues. Therefore, it will be important to develop a long-term time-lapse imaging system for living HIV-1 infected CD4^+^ T cells to fully understand how Vpr contributes to HIV pathogenesis.

## Materials and Methods

### Cell Culture and Plasmid Transfection

Human cervical HeLa cells, HeLa/Fucci2 cells [33] and 293 cells were grown in Dulbecco’s modified Eagle medium (DMEM; Invitrogen, Carlsbad, CA) supplemented with 10% heat-inactivated FBS (Sigma-Aldrich, St. Louis, MO). For synchronization of the cell cycle, HeLa/Fucci2 cells were grown in DMEM containing 0.3% FBS. Plasmid transfection was performed using FuGENE HD (Promega, Medison, WI) and Lipofectamine LTX (Invitrogen).

### Plasmid Construction

The expression vector pME18Neo, pME18Neo-Flag, which contained only the Flag sequence and pME18Neo encoding Flag-tagged wild-type Vpr (pME18Neo-FVpr) or an R80A substitution mutant of Vpr (pME18Neo-R80A) has been described previously [7], [56]. For construction of the vector pME/Flag-Vpr-IRES-ZsGreen1 and the control pME/Flag-IRES-ZsGreen1, a fragment containing IRES sequence and ZsGreen1 coding sequence was amplified by PCR with the primers 5′-CCCAAACTTAAGCTTGGTACCGA-3′ and 5′-TAGCGGCCGCTCAGGGCAAGGCGGAGCCGGAG-3′ using pRetroX-IRES-ZsGreen1 (Clontech Laboratories, Mountain View, CA) as a template. The PCR fragment was subcloned into pME18Neo-FVpr and pME18Neo-Flag at the *Not*Ι site. For construction of pME18Neo/Flag-Vpr-IRES-ECFP and the control pME18Neo/Flag-IRES-ECFP, we first amplified the ECFP coding sequence by PCR with the primers 5′-GATGATAATATGGTGAGCAAGGGCGA-3′ and 5′-AAAACGCGTTTACTTGTACAGCTCGTCCA-3′ using pRSET_B-ECFP_ [36] as a template. Second, we amplified pME18Neo/Flag-Vpr-IRES and pME18Neo/Flag-IRES with deletion of the ZsGreen1 coding sequence by PCR with the primers 5′-AAAACGCGTTCTAGAGAAAAAACCTCCCACAC-3′ and 5′-GTGTTTTTCAAAGGAAAACCACGT-3′ using pME18Neo/Flag-Vpr-IRES-ZsGreen1 as a template. These fragments were digested with *Mlu*Ιl phosphorylated with T4 kinase, and ligated. For construction of pME18Neo/Flag-Vpr-IRES-SCAT3.1 and the control pME18Neo/Flag-IRES-SCAT3.1, we amplified three DNA fragments as follows: (i) pME18Neo/Flag-Vpr-IRES and pME18Neo/Flag-IRES with deletion of the ECFP coding sequence by PCR with the primers 5′-AAAACGCGTTCTAGAGAAAAAACCTCCCACAC-3′ and 5′-AAATCTAGATTTCACGTGTTTTTCAAAGGAAAACCACGT-3′ using pME18Neo/Flag-Vpr-IRES-ECFP and pME18Neo/Flag-IRES-ECFP as templates, respectively; (ii) the cDNAs of C-terminally deleted ECFP mutants fused to the DEVD sequence and NLS sequence were amplified by PCR with the primers 5′-GATGATAATATGAACCTGGTGGACCTCCAA-3′ and 5′-CATGGTACCATCGACCTCATC-3′ using SCAT3.1 [36] as a template; and (iii) the cDNAs of Venus fused to the NES sequence were amplified by PCR with the primers 5′-CGATGAGGTCGATGGTACCA-3′ and 5′-AAAACGCGTCTCTAGATGCATGCTCGAGTTA-3′ using SCAT3.1 [36] as a template. The fragments (i), (ii) and (iii) were digested with *PmaC*ΙmaCa *Mlu*Ιlu*Kpn*ΙpnCa t*Kpn*ΙpnCa *Mlu*Ιl respectively. The digested PCR fragments were mixed together and ligated. For construction of pME18Neo/DD-Vpr, we amplified the DD coding sequence by PCR with the primers 5′-AAACTCGAGATGGGAGTGCAGGTGGAAAC-3′ and 5′-AAAGATATCCTTTCCGGTTTTAGAAGCTCCACA-3′ using pPTuner (Clontech). The fragments were subcloned into pME18Neo-FVpr at the *Xho*I and *Eco*RV sites. For construction of pME/DD-Vpr-IRES-ZsGreen1 and pME18Neo/DD-Vpr-IRES-ECFP, the fragment of *Xho*I and *Eco*RV of pME18Neo/DD-Vpr was excised and subcloned into pME18Neo/Flag-Vpr-IRES-ZsGreen1 and pME18Neo/Flag-Vpr-IRES-ECFP, respectively.

For construction of the vector pAdeno-X/Flag-Vpr-IRES-ZsGreen1, the vectors pShuttle2/Flag-Vpr-IRES-ZsGreen1 and pShuttle2/IRES-ZsGreen1 were first constructed. For construction of the vector pShuttle2/Flag-IRES-ZsGreen1, a fragment containing the IRES sequence and the ZsGreen1 coding sequence was amplified by PCR with the primers 5′-TAATCTAGAGCCCCTCTCCCTCCCCCCCCCCTAA-3′ and 5′-TAGCGGCCGCTCAGGGCAAGGCGGAGCCGGAG-3′ using pRetroX-IRES-ZsGreen1 (Clontech Laboratories) as a template. The PCR fragment was subcloned into pShuttle2 at *Xba*I and *Not*Ιotnd ing pRetroX-IRES-ZsGreethe vector pShuttle2/Flag-Vpr-IRES-ZsGreen1, a fragment containing the Flag-Vpr coding sequence was amplified by PCR with the primers 5′-GAAGCTAGCGACTACAAGGATGACGATGACAAAATCGAACAAGCCCCAGAAGA-3′ and 5′-GCACTAGACTAGGATCTACTGGCTCCAT-3′ using pME18Neo-FVpr as a template. The PCR fragment was subcloned into pShuttle2/IRES-ZsGreen1 at *Nhe*I and *Xba*I sites. For construction of the vector pAdeno-X/Flag-Vpr-IRES-ZsGreen1 and the control vector pAdeno-X/IRES-ZsGreen1, pShuttle2/Flag-Vpr-IRES-ZsGreen1 and pShuttle2/IRES-ZsGreen1 were digested with I-*Ceu*I and Pl-*Sce*I and ligated with predigested Adeno-X viral DNA.

### Generation of Adenoviruses Expressing Vpr and ZsGreen1, and the Infection Protocol

Adenoviruses were constructed using the Adeno-X™ expression system (Clontech Laboratories). Briefly, pAdeno-X/Flag-Vpr-IRES-ZsGreen1 and the control pAdeno-X/IRES-ZsGreen1 were digested with *Pac*I. The linear adenoviral DNA was then transfected into low-passage 293 cells. Adenovirus generation was confirmed by the appearance of a cytopathic effect and by the expression of ZsGreen1 protein. The adenoviruses were harvested and viral titers were determined on low-passage 293 cells using Adeno-X™ Rapid Titer Kit (Clontech Laboratories).

For adenovius infection, target cells were seeded in 24- or 6-well polystyrene plates (TPP Techno Plastic Products AG) or 35 mm glass-bottom dishes (IWAKI, Tokyo, Japan). On the following day, the culture medium was removed and the cells were infected at MOI 50 or 100.

### Western Blotting

Cells were lysed for 30 min on ice in 10 mM Tris-HCl (pH 8.0), 150 mM NaCl, 5 mM EDTA, 1% Triton X-100, and 0.1% sodium dodecyl sulfate (SDS) supplemented with a protease inhibitor cocktail (Roche Diagnostics, Basel, Switzerland). Lysates were mixed with SDS-PAGE sample buffer and boiled for 5 min. Protein concentrations were determined with a BCA protein assay kit (Pierce, Rockford, IL) using bovine serum albumin as a standard. Equal amounts of total protein were examined by Western blot. The following antibodies were used: anti-Flag MAb (M2) and anti-βnactin MAb (Sigma-Aldrich), anti-cleaved caspase-3 (Asp175) polyclonal antibody, anti-caspase-3 polyclonal antibody (Cell Signaling Technology, Beverly, MA), anti-GFP MAb (MBL, Nagoya, Japan), horseradish-peroxidase (HRP)-conjugated goat anti-mouse IgG (Amersham Bioscience, Uppsala, Sweden), and HRP-conjugated goat anti-rabbit IgG (Amersham Bioscience). Signals were visualized after the treatment of the membrane with SuperSignal West Pico chemiluminescent substrate (Pierce).

### Immunofluorescence Staining

HeLa cells on a cover slip were fixed in 3.6% formaldehyde in phosphate-buffered saline (PBS) for 10 min at room temperature and permeabilized with PBS containing 0.2% Triton X-100 for 5 min on ice. After treating with PBS containing 5% skim milk for 15 min at room temperature (RT), cells were stained with the first antibody and the appropriate secondary antibody in PBS containing 2.5% skim milk for 30 min. After rinsing with PBS, the cover slip was mounted on glass slides in PBS containing 90% glycerol and analyzed by confocal laser scanning microscopy (Olympus, FV1000D). A 440 nm excitation laser was used for ECFP and SCAT3.1, and a 559 nm excitation laser was used for Alexa594. The SCAT3.1 Em fluorescence was split by an SDM510 dichroic mirror into 460–500 nm (ECFP) and 515–615 nm (Venus).

### Fluorescence Imaging of HeLa/Fucci2 Cells

HeLa/Fucci2 cells were grown on 35 mm glass-bottom dishes and transfected with the indicated vectors or infected with adenoviral vector expressing Vpr and ZsGreen1 virus. At the indicated times after transfection or infection, cells were subjected to long-term time-lapse imaging using a computer-assisted fluorescence microscope (Olympus, LCV110) equipped with an objective lens (Olympus, UAPO 40X/340 N.A. = 0.90), a halogen lamp, a red LED (620 nm), a CCD camera (Olympus, DP30), and interference filters. For fluorescence imaging of Fucci2 with ECFP or ZsGreen1, the halogen lamp was used with an FF02-510/10-25 excitation filter (Semrock, Inc., Rochester, NY), an FF520-Di02-25x36 dichroic mirror (Semrock), and an FF01-542/27-25 emission filter (Semrock) for Venus; an FF01-562/40-25 excitation filter (Semrock), an FF593-Di03-25x36 dichroic mirror (Semrock), and an FF01-641/75-25 emission filter (Semrock) for mCherry; and a CFP2432B filter cube (Semrock) for ECFP; an FF02-472/30-25 excitation filter (Semrock), and an FF495-Di03-25x36 dichroic mirror (Semrock), and an FF01-520/35-25 emission filter (Semrock) for ZsGreen1. Image acquisition and analysis were performed using MetaMorph 7.7.4 software (Universal Imaging, Media, PA). Movies were assembled using QuickTime software.

### Analysis of Cell Cycle Profiles by CELAVIEW Microscope

HeLa/Fucci2 cells were transfected with pME18Neo-FVpr or pME18Neo-R80A or infected with adenoviral vector expressing Vpr and ZsGreen1 virus and plated in 24-well polystyrene plates (TPP Techno Plastic Products, Cat.92024). At 72 h post-transfection or -infection, the cells were fixed in 3.6% formaldehyde in PBS for 10 min at room temperature and permeabilized with PBS containing 0.2% Triton X-100 for 5 min on ice. After treating with PBS containing 5% skim milk for 15 min at RT, the cells were subjected to immunofluorescence staining with anti-Flag MAb (M2) (Sigma-Aldrich) Alexa680 conjugated anti-mouse IgG (Invitrogen) in PBS containing 2.5% skim milk for 30 min. After rinsing with PBS, the cells were stained with 5 µg/ml Hoechst 33342 (ImmunoChemistry Technologies LLC) for 10 min at RT, and washed with PBS three times. For each sample, at least 200 Alexa680-positive cells were observed and analyzed using a CELAVIEW microscope (OLYMPUS: RS100).

HeLa cells were infected with adenoviral vector expressing Vpr or expressing only ZsGreen1 as a control. At 72 h post-infection, the cells were fixed and stained in 3.6% formaldehyde containing Hoechst 33342 (ImmunoChemistry Technologies LLC) for 10 min at RT, and then washed with PBS three times. For each sample, at least 200 ZsGreen1-positive cells were observed and analyzed using a CELAVIEW microscope (OLYMPUS: RS100).

### Analysis of Cell Cycle Profiles by Flow Cytometry

HeLa cells were transfected with pME18Neo/DD-Vpr-IRES-ZsGreen1 or pME18Neo/DD-IRES-ZsGreen1 as a control and cultured for 24 h. The cells were then cultured in the presence or absence of 500 nM Shield1. Twenty-four hours after Shield1 addition, the cells were harvested and fixed with 1% formaldehyde, followed by 70% ethanol. Fixed cells were incubated in PBS containing RNase A (50 µg/ml) at 37°C for 20 min and then stained with propidium iodide (PI, 40 µg/ml). For each sample, at least 7,000 cells were analyzed using a FACS Calibur instrument (Becton-Dickinson, Mountain View, CA) with the CELL Quest software (Becton-Dickinson). Ratios of the numbers of cells in G1 and G2/M phases (G2/M:G1 ratios) were calculated using ModFit LT Software (Verity Software House, Topsham, ME).

### Fluorescence FRET Imaging of Caspase-3 Activity

HeLa/Fucci2 cells were grown on 35 mm glass-bottom dishes in phenol red-free DMEM containing 10% FBS and transfected with either pME18Neo/Flag-Vpr-IRES-ECFP or the control pME18Neo/Flag-IRES-ECFP. The cells underwent long-term, time-lapse imaging using a computer-assisted fluorescence microscope (Olympus, LCV110) equipped with an objective lens (Olympus, UAPO 40X/340 N.A. = 0.90), a halogen lamp, a red LED (620 nm), a CCD camera (Olympus, DP30), and interference filters. For fluorescence imaging of FRET, we used the halogen lamp with a BP425-445HQ excitation filter (Olympus), a DM450 dichroic mirror (Olympus), and an FF01-542/27-25 emission filter (Semrock) for observing SCAT3.1. To quantify the results, the images of ECFP and Venus fluorescence were processed with MetaMorph 7.7.4 software (Universal Imaging), and the Venus/ECFP emission ratio was calculated.

### Analysis of Apoptosis

To detect active caspase-3-positive apoptotic cells, we used flow cytometry. HeLa cells were transfected with either pME18Neo/DD-IRES-ZsGreen1 or pME18Neo/DD-Vpr-IRES-ZsGreen1. The transfected cells were harvested and stained with SR-DEVD-FMK (ImmunoChemistry Technologies LLC) according to the manufacturer’s protocols. For each sample, 10,000 ZsGreen1-positive cells were analyzed using a FACS Calibur instrument (Becton-Dickinson).

### ProteoTuner Method for Inducible Vpr Expression

Cells were transfected with the indicated vectors. Twenty-four hours after transfection, Shield1 was added to the culture medium at 500 nM to induce Vpr expression in the cells.

### Statistical Analysis

The two-tailed t-test was used for statistical determinations. P values <0.05 were considered statistically significant.

## Supporting Information

Figure S1 **The localization of Flag-Vpr and ECFP.** HeLa cells were transfected with pME18Neo/Flag-Vpr-IRES-ECFP or the control pME18Neo/Flag-IRES-ECFP. At 24 h after transfection, cells were stained with anti-Flag MAb M2 followed by Alexa594 conjugated anti-mouse IgG MAb and analyzed by confocal laser scanning microscopy. Cells showing red and cyan fluorescence express Flag-Vpr and ECFP, respectively. The scale bar represents 10 µm.
(TIF)

Figure S2 **The expression of Flag-Vpr and ECFP.** HeLa/Fucci2 cells were transfected with pME18Neo/Flag-Vpr-IRES-ECFP or the control pME18Neo/Flag-IRES-ECFP. At 24 h after transfection, cells were lysed and subjected to Western blot analysis with anti-Flag MAb M2, anti-GFP MAb, and anti-β-Actin MAb.
(TIF)

Figure S3 **The percentages of ECFP-expressing cells observed during time-lapse imaging.** HeLa/Fucci2 cells were transfected with pME/Flag-Vpr-IRES-ECFP or pME/Flag-IRES-ECFP as a control. Twenty-four hours after transfection, ECFP-expressing cells were observed under an incubator fluorescence microscope (Olympus: LCV110) at 15 min intervals for 72 h. The percentages of ECFP-expressing cells were counted in particular areas at 24, 36, 48, 60, 72, 84, and 96 h post-transfection (Figure 2).
(TIF)

Figure S4 **The localization of Flag-Vpr and SCAT3.1.** HeLa cells were transfected with pME18Neo/Flag-Vpr-IRES-SCAT3.1 or the control pME18Neo/Flag-IRES-SCAT3.1. At 24 h after transfection, cells were fixed, permeabilized, stained with anti-Flag MAb M2 followed by Alexa594 conjugated anti-mouse IgG MAb, and analyzed by confocal laser scanning microscopy. The Alexa594 fluorescence images (red) and the SCAT3.1 fluorescence images (cyan and yellow) were acquired using 559 and 440 nm excitation lasers, respectively. The SCAT3.1 emission fluorescence was split by an SDM510 dichroic mirror into two: 460–500 nm (ECFP) and 515–615 nm (Venus). The scale bar represents 10 µm.
(TIF)

Figure S5 **The expression of Flag-Vpr and SCAT3.1.** HeLa/Fucci2 cells were transfected with pME18Neo/Flag-Vpr-IRES-SCAT3.1 or the control pME18Neo/Flag-IRES-SCAT3.1. At 24, 48, 72, and 96 h after transfection, cells were lysed and subjected to Western blot analysis with anti-Flag MAb, anti-GFP MAb, and anti-β-actin MAb.
(TIF)

Figure S6 **Vpr induces apoptosis via caspase-3 activation.** The time course of the 530/480 emission ratio from 26 h to 33 h post-transfection in #3 cells and in #2 cells as a control (Figure 4). We analyzed the 530 nm fluorescence intensity of SCAT3.1 and the 480 nm fluorescence intensity of ECFP in the cytoplasm, and calculated the 530/480 emission ratio using MetaMorph 7.7.4 software.
(TIF)

Video S1 **Time-lapse imaging of cell cycle progression in ECFP-positive HeLa/Fucci2 cells.** HeLa/Fucci2 cells were transfected with pME/Flag-IRES-ECFP. Twenty-four hours after transfection, ECFP-expressing cells were observed with LCV110 Imaging System at 15 min intervals for 72 h.
(MP4)

Video S2 **Time-lapse imaging of Vpr-induced cell cycle arrest and cell death in HeLa/Fucci2 cells.** HeLa/Fucci2 cells were transfected with pME/Flag-Vpr-IRES-ECFP. Twenty-four hours after transfection, ECFP-expressing cells were observed with the LCV110 Imaging System at 15 min intervals for 72 h.
(MP4)

Video S3 **Time-lapse imaging of cell cycle progression in SCAT3.1-expressing HeLa/Fucci2 cells.** HeLa/Fucci2 cells were transfected with pME/Flag-IRES-SCAT3.1. Twenty-four hours after transfection, SCAT3.1-expressing cells were observed with LCV110 Imaging System at 15 min intervals for 72 h.
(MP4)

Video S4 **Time-lapse imaging of Vpr-induced G2 arrest and caspase-3-dependent apoptosis in HeLa/Fucci2 cells.** HeLa/Fucci2 cells were transfected with pME/Flag-Vpr-IRES-SCAT3.1. Twenty-four hours after transfection, ECFP-expressing cells were observed with LCV110 Imaging System at 15 min intervals for 72 h.
(MOV)

Video S5 **Time-lapse imaging of the cell cycle progression in untreated ECFP-positive HeLa/Fucci2 cells with Shield1.** HeLa/Fucci2 cells were transfected with pME/DD-Vpr-IRES-ECFP and cultured. Forty-eight hours after transfection, the cells were washed and further cultured in the absence of 500 nM Shield1 for 1 h. ECFP-expressing cells were observed with the LCV110 Imaging System at 15 min intervals for 72 h.
(MP4)

Video S6 **Time-lapse imaging of the effect of transient expression of DD-Vpr in HeLa/Fucci2 cells.** HeLa/Fucci2 cells were transfected with pME/DD-Vpr-IRES-ECFP and cultured. Twenty-four hours after transfection, the cells were treated with 500 nM Shield1 for 23 h, at which point the cells were washed to remove Shield1 and further cultured in the absence of 500 nM Shield1 for 1 h. ECFP-expressing cells were observed with LCV110 Imaging System at 15 min intervals for 72 h.
(MP4)

Video S7 **Time-lapse imaging of the effect of continuous expression of DD-Vpr in HeLa/Fucci2 cells.** HeLa/Fucci2 cells were transfected with pME/DD-Vpr-IRES-ECFP. Twenty-four hours after transfection, the cells were treated with 500 nM Shield1 for 23 h, at which point the cells were washed to remove Shield1 and further cultured in the presence of 500 nM Shield1 for 1 h. ECFP-expressing cells were observed with LCV110 Imaging System at 15 min intervals for 72 h.
(MP4)
