# Supplementary figures and images for: Visualizing Vpr-Induced G2 Arrest and Apoptosis
Source: PLoS One. 2014 Jan 22;9(1):e86840. doi: 10.1371/journal.pone.0086840 (PMC3899331; doi:10.1371/journal.pone.0086840)

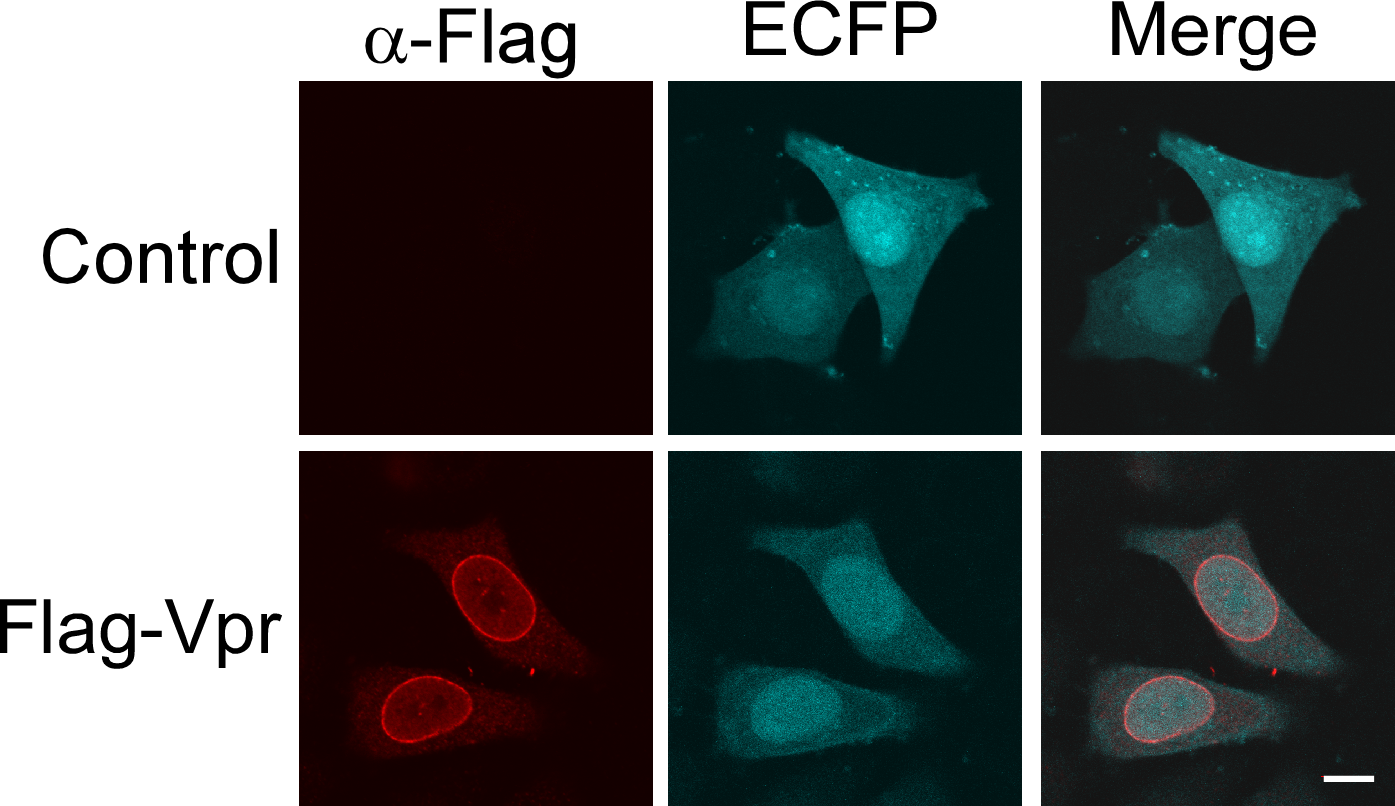

Supplement: Figure S1 — The localization of Flag-Vpr and ECFP. HeLa cells were transfected with pME18Neo/Flag-Vpr-IRES-ECFP or the control pME18Neo/Flag-IRES-ECFP. At 24 h after transfection, cells were stained with anti-Flag MAb M2 followed by Alexa594 conjugated anti-mouse IgG MAb and analyzed by confocal laser scanning microscopy. Cells showing red and cyan fluorescence express Flag-Vpr and ECFP, respectively. The scale bar represents 10 µm. (TIF) [file pone.0086840.s001.tif]

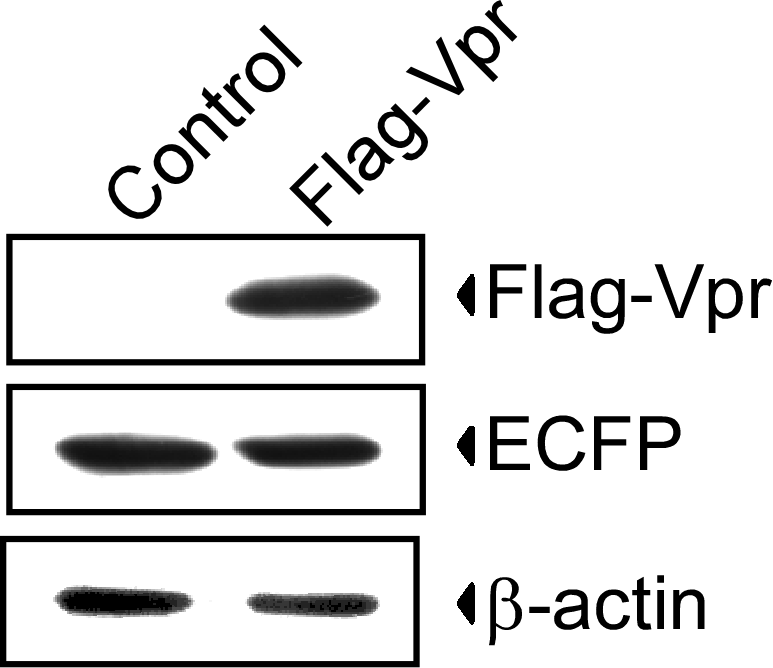

Supplement: Figure S2 — The expression of Flag-Vpr and ECFP. HeLa/Fucci2 cells were transfected with pME18Neo/Flag-Vpr-IRES-ECFP or the control pME18Neo/Flag-IRES-ECFP. At 24 h after transfection, cells were lysed and subjected to Western blot analysis with anti-Flag MAb M2, anti-GFP MAb, and anti-β-Actin MAb. (TIF) [file pone.0086840.s002.tif]

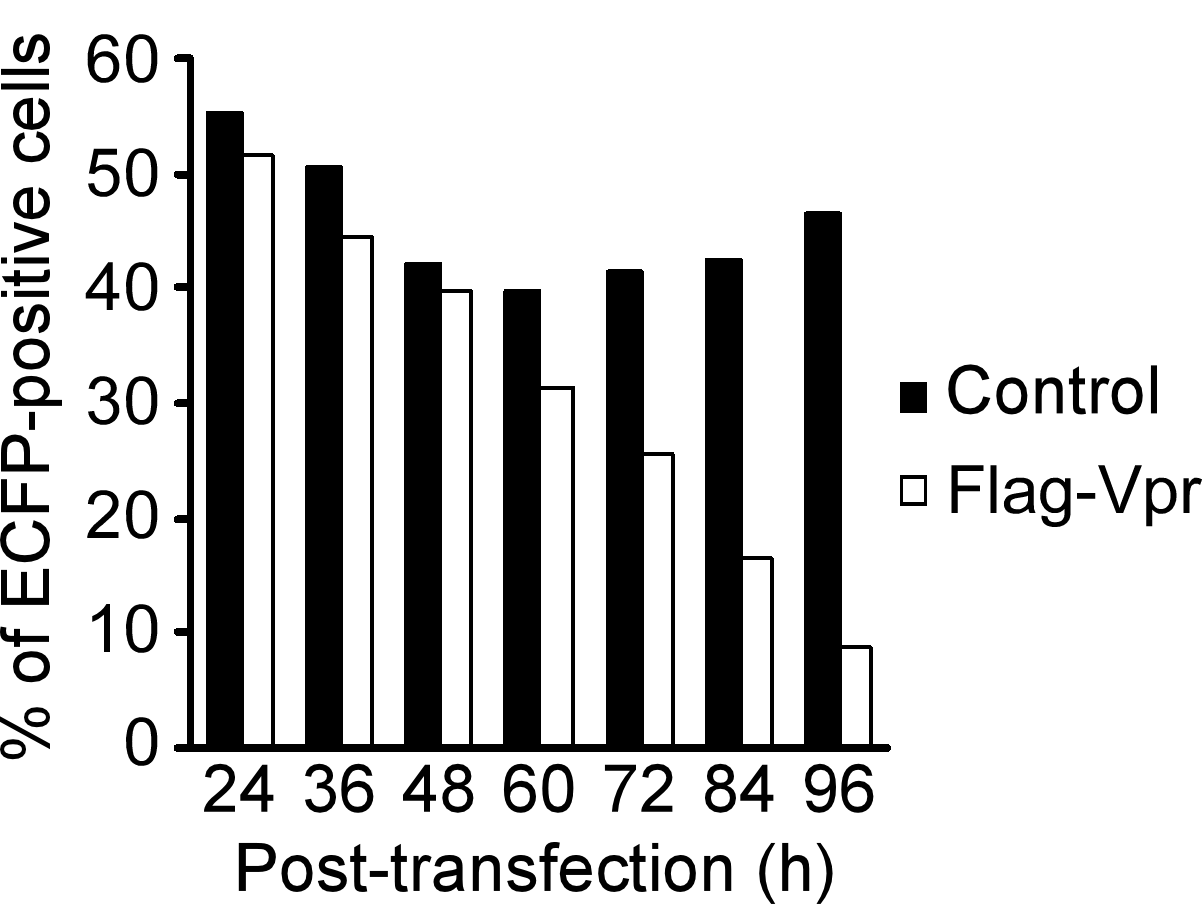

Supplement: Figure S3 — The percentages of ECFP-expressing cells observed during time-lapse imaging. HeLa/Fucci2 cells were transfected with pME/Flag-Vpr-IRES-ECFP or pME/Flag-IRES-ECFP as a control. Twenty-four hours after transfection, ECFP-expressing cells were observed under an incubator fluorescence microscope (Olympus: LCV110) at 15 min intervals for 72 h. The percentages of ECFP-expressing cells were counted in particular areas at 24, 36, 48, 60, 72, 84, and 96 h post-transfection (Figure 2). (TIF) [file pone.0086840.s003.tif]

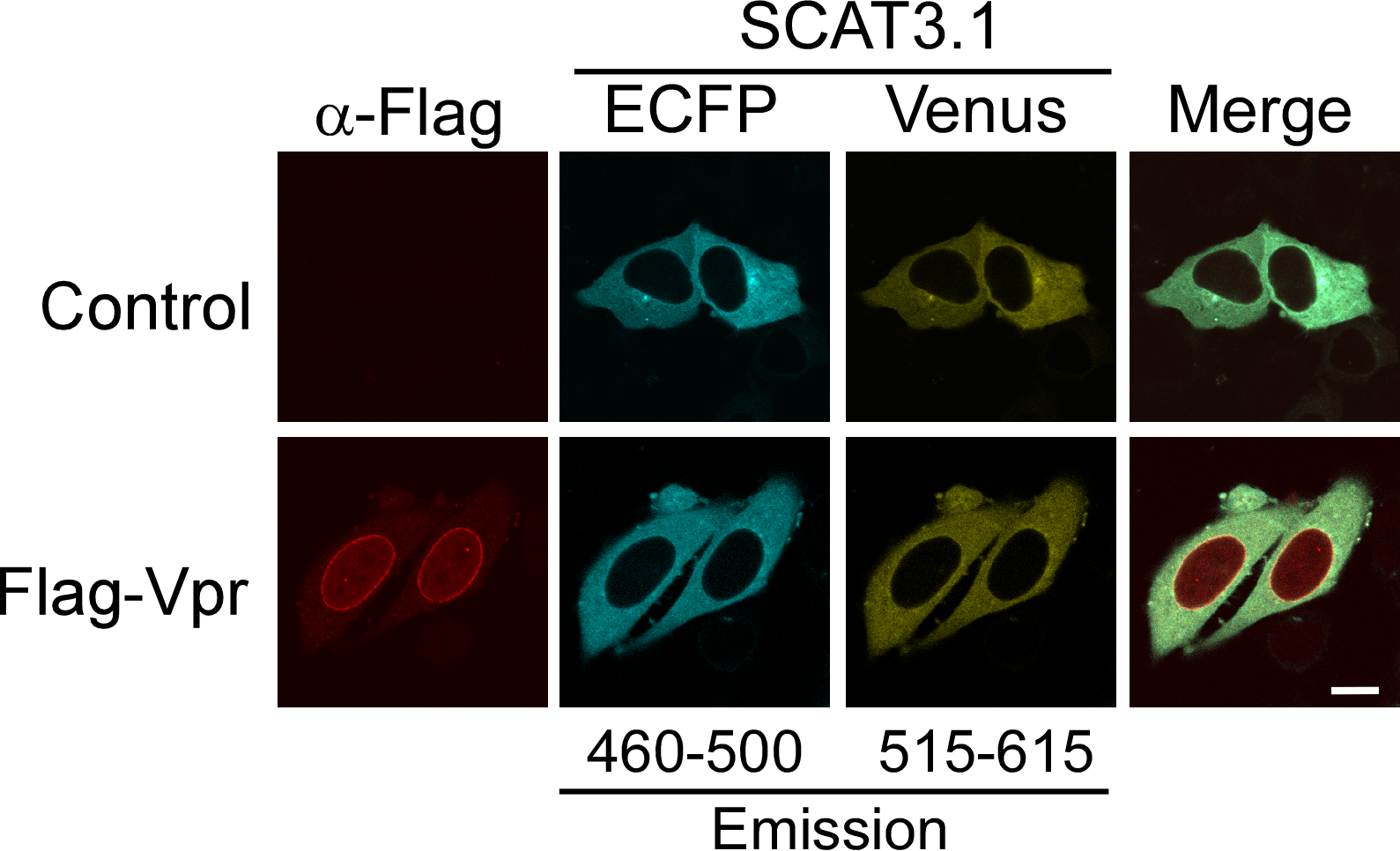

Supplement: Figure S4 — The localization of Flag-Vpr and SCAT3.1. HeLa cells were transfected with pME18Neo/Flag-Vpr-IRES-SCAT3.1 or the control pME18Neo/Flag-IRES-SCAT3.1. At 24 h after transfection, cells were fixed, permeabilized, stained with anti-Flag MAb M2 followed by Alexa594 conjugated anti-mouse IgG MAb, and analyzed by confocal laser scanning microscopy. The Alexa594 fluorescence images (red) and the SCAT3.1 fluorescence images (cyan and yellow) were acquired using 559 and 440 nm excitation lasers, respectively. The SCAT3.1 emission fluorescence was split by an SDM510 dichroic mirror into two: 460–500 nm (ECFP) and 515–615 nm (Venus). The scale bar represents 10 µm. (TIF) [file pone.0086840.s004.tif]

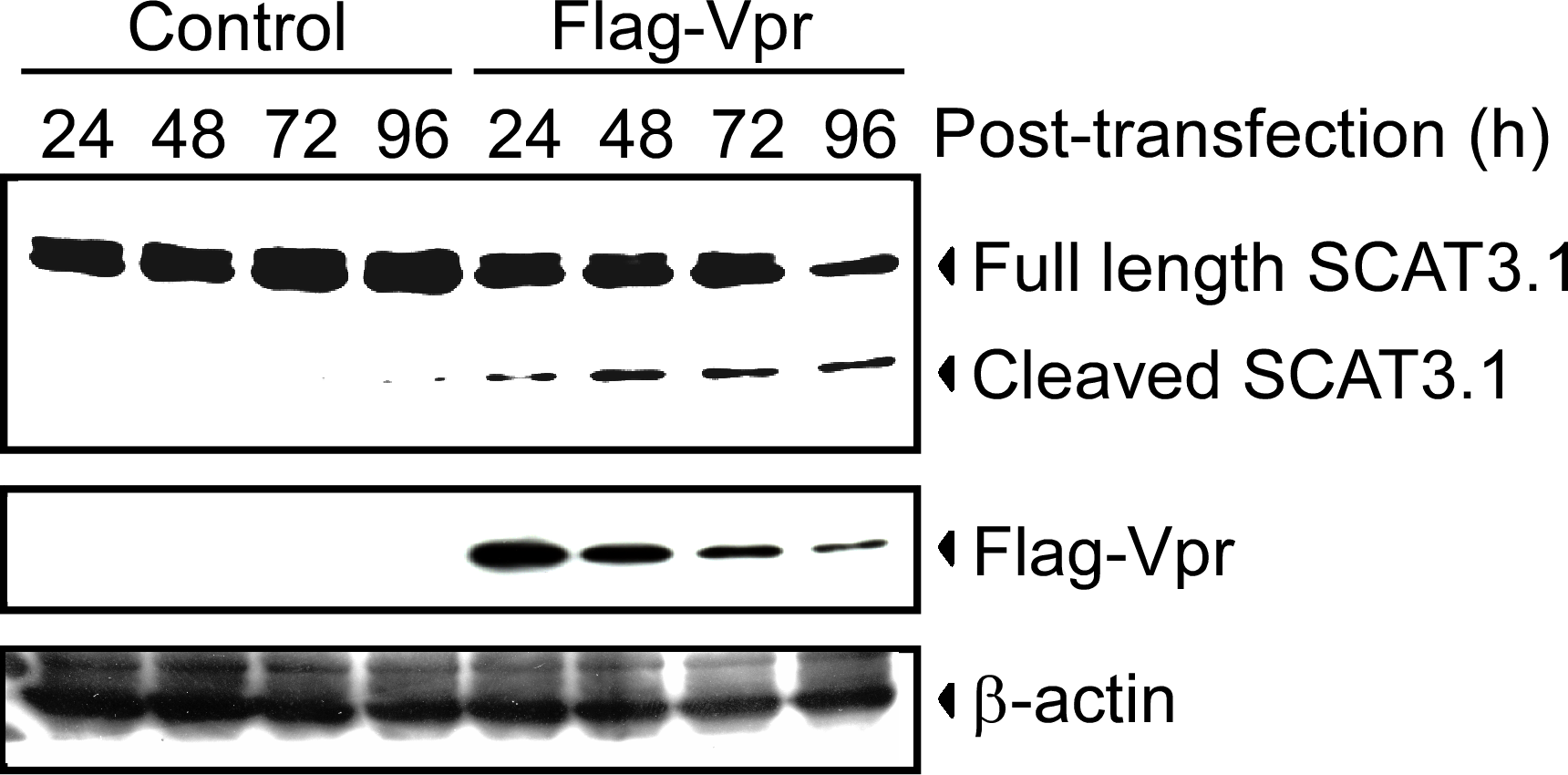

Supplement: Figure S5 — The expression of Flag-Vpr and SCAT3.1. HeLa/Fucci2 cells were transfected with pME18Neo/Flag-Vpr-IRES-SCAT3.1 or the control pME18Neo/Flag-IRES-SCAT3.1. At 24, 48, 72, and 96 h after transfection, cells were lysed and subjected to Western blot analysis with anti-Flag MAb, anti-GFP MAb, and anti-β-actin MAb. (TIF) [file pone.0086840.s005.tif]

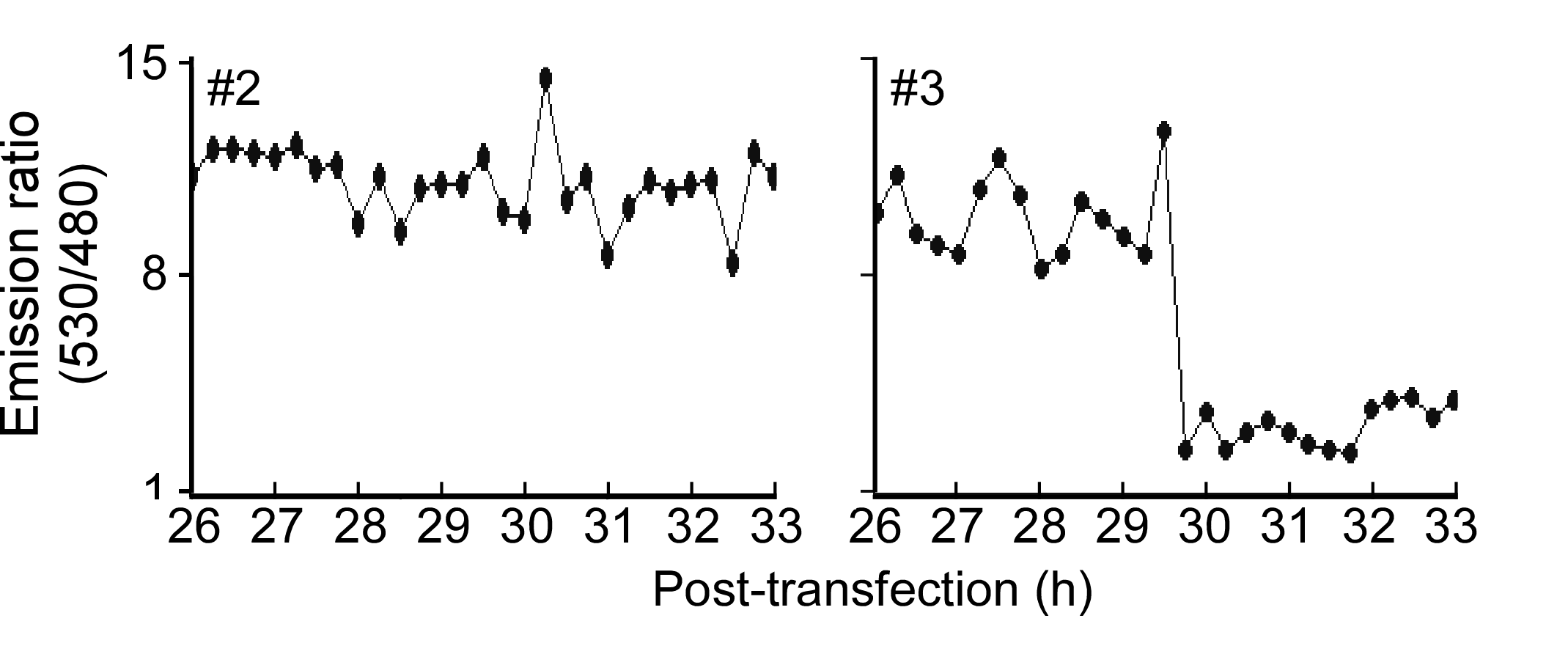

Supplement: Figure S6 — Vpr induces apoptosis via caspase-3 activation. The time course of the 530/480 emission ratio from 26 h to 33 h post-transfection in #3 cells and in #2 cells as a control (Figure 4). We analyzed the 530 nm fluorescence intensity of SCAT3.1 and the 480 nm fluorescence intensity of ECFP in the cytoplasm, and calculated the 530/480 emission ratio using MetaMorph 7.7.4 software. (TIF) [file pone.0086840.s006.tif]
